# Aerobic glycolysis enhances HBx-initiated hepatocellular carcinogenesis via NF-κBp65/HK2 signalling

**DOI:** 10.1186/s13046-022-02531-x

**Published:** 2022-11-21

**Authors:** Lingjun Chen, Xianyi Lin, Yiming Lei, Xuan Xu, Qi Zhou, Yan Chen, Huiling Liu, Jie Jiang, Yidong Yang, Fengping Zheng, Bin Wu

**Affiliations:** 1 Department of Gastroenterology, The Third Affiliated Hospital of Sun Yat-Sen University, Guangzhou, 510630 Guangdong Province China; 2 Guangdong Provincial Key Laboratory of Liver Disease Research, Guangzhou, 510630 Guangdong Province China

**Keywords:** Hepatocellular carcinoma, Hepatitis B virus X protein, NF-κBp65, Aerobic glycolysis, Hexokinase 2

## Abstract

**Background:**

Aerobic glycolysis has been recognized as one of the growth-promoting metabolic alterations of cancer cells. Emerging evidence indicates that nuclear factor κB (NF-κB) plays significant roles in metabolic adaptation in normal cells and cancer cells. However, whether and how NF-κB regulates metabolic reprogramming in hepatocellular carcinoma (HCC), specifically hepatitis B virus X protein (HBx)-initiated HCC, has not been determined.

**Methods:**

A dataset of the HCC cohort from the TCGA database was used to analyse the expression of NF-κB family members. Expression of NF-κBp65 and phosphorylation of NF-κBp65 (p-p65) were detected in liver tissues from HBV-related HCC patients and normal controls. A newly established *HBx*^+*/*+^/*NF-κBp65*^*f/f*^ and *HBx*^+*/*+^*/NF-κBp65*^*Δhepa*^ spontaneous HCC mouse model was used to investigate the effects of NF-κBp65 on HBx-initiated hepatocarcinogenesis. Whether and how NF-κBp65 is involved in aerobic glycolysis induced by HBx in hepatocellular carcinogenesis were analysed in vitro and in vivo.

**Results:**

NF-κBp65 was upregulated in HBV-related HCC, and HBx induced NF-κBp65 upregulation and phosphorylation in vivo and in vitro. Hepatocyte-specific *NF-κBp65* deficiency remarkably decreased HBx-initiated spontaneous HCC incidence in *HBx*-TG mice. Mechanistically, HBx induced aerobic glycolysis by activating NF-κBp65/hexokinase 2 (HK2) signalling in spontaneous hepatocarcinogenesis, and overproduced lactate significantly promoted HCC cell pernicious proliferation via the PI3K (phosphatidylinositide 3-kinase)/Akt pathway in hepatocarcinogenesis.

**Conclusion:**

The data elucidate that NF-κBp65 plays a pivotal role in HBx-initiated spontaneous HCC, which depends on hyperactive NF-κBp65/HK2-mediated aerobic glycolysis to activate PI3K/Akt signalling. Thus, phosphorylation of NF-κBp65 will be a potential therapeutic target for HBV-related HCC.

**Supplementary Information:**

The online version contains supplementary material available at 10.1186/s13046-022-02531-x.

## Background

Hepatocellular carcinoma (HCC), the most common type of primary liver cancer, is the third leading cause of cancer-related death in the world according to the latest statistics [1]. Chronic infection with hepatitis B virus (HBV) is one of the most frequent risk factors for HCC [2]. The HBV genome encodes four viral gene proteins, one of which is Hepatitis B virus X protein (HBx). HBx is a 17 kDa multifunctional protein and is essential for HBV replication and the initiation and development of HCC [3–7]. Recently, emerging studies revealed that HBx is closely involved in aerobic glycolysis in hepatocarcinogenesis [8–11]. Aerobic glycolysis, also termed the Warburg effect, is considered a hallmark feature of cancer. Unlike normal cells that process glucose into carbon dioxide by oxidative phosphorylation (OXPHOS) in the mitochondria, cancer cells prefer to metabolize glucose into lactate in the cytoplasm even when oxygen is sufficient [12, 13]. To compensate for the lower energy production by aerobic glycolysis compared to OXPHOS, cancer cells increase glucose uptake by upregulating glucose transporters, prominently glucose transporter 1 (GLUT1) and the majority of glycolytic enzymes, such as hexokinase 2 (HK2) and lactate dehydrogenase A (LDHA) [14].

It has been shown that aerobic glycolysis increases in HBV-associated HCC [15, 16], and metabonomic analysis showed that multiple metabolites associated with aerobic glycolysis were increased in HBV or HBx transfection in primary rat hepatocytes [8]. Furthermore, HBx induced BNIP3L-dependent mitophagy to upregulate aerobic glycolysis, increasing cancer stemness in hepatocarcinogenesis [11]. However, the specific mechanism by which HBx induces aerobic glycolysis is largely unclear. Therefore, the regulatory mechanism of HBx-induced glycolysis merits further investigation, and it would be helpful to identify a novel therapeutic target.

The nuclear factor κB (NF-κB) family comprises five transcription factors, named RelA (p65), RelB, c-Rel, NF-κB1 (p105/p50) and NF-κB2 (p100/p52) [17]. All five proteins form homo or heterodimers in the cytoplasm as an inactive complex combined with inhibitory molecules called IκB proteins in the resting state. IκBs are regulated by IκB kinase (IKK), which consists of two catalytic subunits, IKK-α and IKK-β, and a regulatory subunit, IKK-γ/NEMO (NF-κB essential modulator) [18]. Activation of NF-κB pathways mainly consists of canonical and noncanonical pathways. The canonical pathway is primarily activated by phosphorylation of IKK, which can then cause IκB protein phosphorylation and ubiquitin-mediated proteasome degradation, allowing NF-κB to translocate to the nucleus to activate target gene expression [19]. The NF-κB pathway plays an essential role in innate immunity, inflammation, cell proliferation, differentiation and metabolic reprogramming [20–23]. We have previously reported that phosphorylation of NF-κBp65 drives hepatocellular tumorigenesis [24]. Several studies have demonstrated that activation of canonical NF-κB signalling drives aerobic glycolysis in sarcoma [25] and central nervous system lymphoma [26]. However, the effect of NF-κBp65 on glycolytic metabolism in HBx-initiated HCC has not been investigated.

Akt, also known as protein kinase B or PKB, is a serine/threonine protein kinase that regulates diverse cellular functions, including metabolism, cell growth and proliferation, through phosphatidylinositol 3-kinase (PI3K) [27]. PI3K/Akt can be activated in response to lactic acid and can promote angiogenesis in endothelial cells [28]. Dysregulation of the PI3K/Akt pathway is frequently implicated in cancers, including HCC [29]. The mechanism of lactate-induced Akt activation in HCC cells is still unclear, and the interaction between lactate and Akt in HCC needs further investigation.

In this study, we explored the interaction between NF-κBp65-mediated aerobic glycolysis and pernicious proliferation in HBx-initiated HCC. Our results demonstrate that HBx increases NF-κBp65 expression and phosphorylation and that hepatocyte-specific *NF-κBp65* deficiency suppresses HBx-induced aerobic glycolysis and subsequent pernicious proliferation, resulting in less carcinogenesis. These results indicate that HBx induces aerobic glycolysis via the NF-κBp65/HK2 pathway to overproduce lactate and that lactate activates PI3K/Akt signalling to enhance hepatocyte pernicious proliferation, resulting in HBx-initiated hepatocellular carcinogenesis.

## Materials and methods

### Clinical tissue samples

Ten normal liver tissue samples were obtained from the adjacent sites of haemangioma patients without hepatitis, and 10 liver specimens from HCC patients with hepatitis B virus infection were obtained during operations before any therapeutic intervention at the Third Affiliated Hospital of Sun Yat-Sen University (Table S1). All the samples were subsequently verified by histology. Informed consent was signed by all the patients prior to the surgery. The acquisition of these liver tissue samples was approved by the Clinical Research Ethics Committee of The Third Affiliated Hospital of Sun Yat-Sen University ([2014] 2–7). The Gene Expression Profiling Interactive Analysis (GEPIA) website (http://gepia.cancer-pku.cn/) was used for the survival analysis of HCC patients based on The Cancer Genome Atlas (TCGA) datasets.

Tissue microarrays of human liver tumours and paired adjacent normal tissues (TFHCC-02, TFHCC-03) were purchased from Shanghai Tufei Biotech (Table S2). Immunohistochemical (IHC) staining was performed on tissue microarrays as described in our previous study [29]. The immunoreactivity score (IRS) gives a range of 0–12 as a product of multiplication between the positive cell proportion score (0–4) and the staining intensity score (0–3) [30]. For statistical analysis, scores of 0 to 7 were considered weak expression, and scores of 8 to 12 were considered strong expression. All experiments were performed with the approval of the Third Affiliated of Sun Yat-sen University of Medicine Review Board.

### Mice

Animal experiments were approved by the Institutional Animal Care and Use Committee of the Third Affiliated Hospital of Sun Yat-Sen University. *HBx*^+/+^ and WT (*HBx*^−/−^) littermates on a mixed genetic background (C57BL/6 and CBA) were generated from *HBx* heterozygous transgenic male and female mice (kindly provided by Dr. DY Yu, Korea Research Institution of Bioscience and Biotechnology, Korea). Mice carrying the LoxP-flanked *NF-κBp65* allele (*NF-κBp65*^*flox/flox*^) were kindly provided by Dr. Jianping Ye (Pennington Biomedical Research Center, Louisiana State University System, Baton Rouge, LA, USA). *Alb*-cre transgenic mice were purchased from The Jackson Laboratory. Mice with hepatocyte-specific *NF-κBp65* deletion (*NF-κBp65*^*Δhepa*^) were generated by crossing *NF-κBp65*^*flox/flox*^ mice with *Alb*-cre transgenic mice, which show hepatocyte-specific expression of Cre recombinase and *NF-κBp65* ablated solely in hepatocytes but not in nonparenchymal liver cells. *NF-κBp65*^*f/f*^ was used as wild-type (WT) mice. *HBx*^+*/*+^*/NF-κBp65*^*f/f*^ mice were generated by crossing *HBx*^+*/*+^ and *NF-κBp65*^*f/f*^ littermates, and *HBx*^+*/*+^*/NF-κBp65*^*Δhepa*^ mice were generated from *HBx*^+*/*+^ and *NF-κBp65*^*Δhepa*^ mice. Genotyping was performed as previously described [31, 32]. Male mice were used in the experiment. For the spontaneous HCC model, WT, *HBx*^+*/*+^*/NF-κBp65*^*f/f*^ and *HBx*^+*/*+^*/NF-κBp65*^*Δhepa*^ mice were fed until 18 months. Six-month-old and 12-month-old mice were sacrificed (*n* = 6 in each group). The 18-month-old mice were sacrificed after ultrasonography (*n* = 6 in the WT group, *n* = 24 in HBx^ + / +^ /NF-κBp65^f/f^ and HBx^ + / +^ /NF-κBp65^Δhepa^). The sedative consisted of xylazine (15 mg/kg), and ketamine (50 mg/kg) was given intraperitoneally as the anaesthesia. Carbon dioxide inhalation was used as a method of euthanasia. The number and size of tumour nodules in the liver lobe were recorded. The liver weight and the mouse gross weight were also measured and recorded.

### Ultrasonography in a spontaneous HCC mouse model

Liver tumour formation was measured by using a preclinical ultrasound imaging system (Vevo 3100; Fujifilm Visual Sonics, Inc.) with an MX250 high-frequency linear array transducer (frequency of 21 MHz) on a digitized scale. Eighteen-month-old mice were anaesthetized with isoflurane at room temperature. Hair removal cream was used to depilate the abdomen of the mice. Ultrasound examination was used to detect liver tumour incidence and size.

### Sample collection

Immediately after the mice were sacrificed, the entire liver was carefully removed and rinsed thoroughly with ice-cold physiological saline. The liver tissues were harvested and stored at − 80 °C before protein, mRNA and biochemical analyses. Part of the liver lobe was fixed in neutral buffered formalin at room temperature to prepare paraffin sections. Liver specimens of hepatic haemangioma and HCC patients were processed in a similar way.

### Haematoxylin and eosin staining, immunohistological staining and immunofluorescence staining

Haematoxylin and eosin (H&E) staining and immunohistochemical (IHC) staining were performed on paraffin sections as described in our previous study [29]. Immuno-fluorescence (IF) staining of cells was also performed as previously described [29]. Semiquantitative analysis of the histological staining was performed using ImageJ software. IHC and IF staining were performed by using antibodies against HBx (1:200, 22,741, Genetex), NF-κBp65 (1:200, 8242, CST), p-p65 (1:200, SAB5700363, Sigma‒Aldrich, 1:200, 3033, CST), Ki-67 (1:200, ab15580, Abcam), Glut1 (1:200, 12939S, CST), HK2 (1:200, ab209847, Abcam), and LDHA (1:200, 3582 T, CST).

### Cell culture and treatments

HepG2, HepG2.2.15, Hep3B (HCC cell lines), HepG2 expressing sodium taurocholate cotransporting polypeptide (HepG2-NTCP), which has been identified as a functional receptor for HBV, and LO2 (human normal hepatocyte cell line) were used in this study. HepG2 and Hep3B cell lines were purchased from the American Type Culture Collection (ATCC, Manassas, VA, USA). LO2, HepG2-NTCP and HepG2.2.15 cells were obtained from the Guangdong Provincial Key Laboratory of Liver Disease Research, China. The cells were cultured in Dulbecco’s modified Eagle’s medium (DMEM) (Gibco BRL, Rockville, MD, USA) for the HepG2, HepG2-NTCP, HepG2.2.15 and Hep3B cell lines or RPMI 1640 medium (Gibco BRL, Rockville, MD, USA) for the LO2 cell line with 10% foetal bovine serum. Cells were treated with Bay 11–7082 (10 μM, Sigma, St Louis, MO, USA), 2-deoxy-D-glucose (2-DG, 10 mM, Selleck Chemicals, Houston, TX, USA), lactate (Sigma, St Louis, MO, USA), sodium pyruvate (Sigma, St Louis, MO, USA), α-cyano-4-hydroxycinnamate (CHC, 5 mM, Selleck Chemicals) or MK-2206 2HCl (10 μM, Selleck Chemicals, Houston, TX, USA).

### Plasmid construction, lentiviral transduction and RNA interference

The plasmid with the 1.3-mer HBV genomic DNA and the HBV 1.3-mer X-null replicon was kindly provided by Wang-Shick Ryu (Addgene, 65,461). Plasmids with pCMV-MCS-NF-κBp65-flag or pCMV-MCS-3flag and plasmids with pcDNA3.1-HBx-HA or pcDNA3.1-HA were constructed. The lentiviral vector encoding the full-length HBx and an HA-tag was purchased from GenePhama (Shanghai, China). The vector alone served as a negative control. Small interfering RNA *siNF-κBp65* (5’-GCACCAUCAACUAUGAUGATT-3’) and *siHK2* (5’-CACGATGAAATTGAACC-TGGT-3’) were purchased from GenePhama (Shanghai, China). Transient transfection, lentiviral transduction and siRNA transfection were conducted using Lipofectamine 3000 transfection reagent (Invitrogen, Carlsbad, CA, USA). We obtained stable HBx-expressing LO2 and HepG2 cell lines by zeocin selection (1 μg/ml) for 14 days before the following experiments.

### Cell viability and growth assay

Cell viability and growth were determined by CCK8 assay (Dojindo Laboratories, Kumamoto, Japan). Briefly, in the cell growth assay, the cells (1 × 10^3^/well) with 100 µl of complete medium were plated into 96-well plates overnight, treated with the corresponding treatment and incubated at 37 °C and 5% CO_2_ for 24 h to 96 h. In the cell viability assay, the cells (5 × 10^3^/well) with 100 µl of complete medium were seeded into 96-well plates overnight and then treated with relevant reagents, such as 2-DG (10 mM) and MK-2206 2HCl (Akt inhibitor, 10 µM). At specified time points, CCK8 reagent (10 μl/well) was added to the wells and incubated at 37 °C for 2 h. Then, the absorbance was measured at a 450-nm wavelength using a microplate reader (Bio Tek-Epoch2, Winooski, VT, USA).

### EdU assay

EdU assays were performed using the Cell-Light EdU Apollo643 In Vitro Kit (RiboBio, Guangzhou, Guangdong Province, China) according to the manufacturer’s protocol. The nuclei of proliferative cells were dyed red. The EdU index was determined by dividing the number of red nuclei cells by the total number of cells in at least 10 randomly selected fields (× 200).

### Isolation of Nuclear-Cytosolic Fractions

The Nuclear and Cytoplasmic Protein Extraction Kit (Beyotime, Shanghai, China) was applied for nuclear and cytosolic fraction separation according to the manufacturer’s instructions. The isolated proteins were quantified by the Pierce BCA Protein Assay Kit (Thermo Fisher, MA, USA) and prepared for western blotting. Histone 3 and β-actin were used as loading controls for the nuclear and cytoplasmic fractions, respectively.

### Western blotting

Total protein extractions were analysed by western blotting as previously described [33]. Western blot bands were visualized using a ChemiDoc imaging system (Bio-Rad, Hercules, CA, USA). Primary antibodies against HBx (1:500, 22,741, Genetex), NF-κBp65 (1:1000, 8242, CST), p-p65 (1:1000, 3033, CST), IKBα (1:1000, 4812, CST), p-IKBα (1:1000, 2859, CST), PCNA (1:2000, 13,110, CST), HA (1:1000, 3724, CST), Flag (1:1000, F1804, Sigma‒Aldrich), histone 3 (1:2000, 4499, CST), Glut1 (1:1000, 12939S, CST), HK2 (1:1000, ab209847, Abcam), LDHA (1:1000, 3582 T, CST), PI3K (1:1000, 4249, CST), Akt (1:1000, 9272, CST), p-Akt (1:1000, 4060, CST), and β-actin (1:3000, A5441, Sigma‒Aldrich) were used. Goat anti-mouse (1:5000, 7076, CST) or goat anti-rabbit (1:5000, 7074, CST) HRP-linked antibodies were used as secondary antibodies. The blot densities were quantified by ImageJ software, and the results were expressed as normalized ratios to the densitometry units of β-actin.

### RNA extraction and PCR assays

Reverse transcription was conducted using SuperScript IV Reverse Transcriptase (Invitrogen, Carlsbad, CA, USA). qPCR was performed using a QuantiTect SYBR Green PCR Kit (Qiagen, Chatsworth, CA, USA). Total RNA was isolated from cells or liver tissues using the RNAgents Total RNA Isolation System (Promega, Madison, WI, USA) according to the manufacturer’s protocol. Reverse transcription was conducted using a Reverse Transcription Kit (TOYOBO, Japan) according to the manufacturer’s instructions. qPCR was performed using a Mini Opticon Real-time PCR System (Bio-Rad, Hercules, CA, USA) with SYBR Green (Invitrogen, Carlsbad, CA, USA). β-actin served as an internal control for qRT‒PCR. Data were calculated using the 2^−ΔΔCT^ method. The primers are listed in Table S3.

### Glucose and lactate measurement

A glucose measurement kit (Nanjing Jian Cheng Bioengineering Institute) was used to measure the glucose concentration in the cell culture medium and fresh liver tissue homogenate. Lactate production in the cell culture medium and the fresh liver tissue homogenate was measured using a lactate assay kit (Nanjing Jian Cheng Bioengineering Institute). All these assays were performed according to the manufacturer’s protocols. The protein concentration of cells or tissues was detected to normalize the glucose and lactate levels.

### ATP Content Assay

Intracellular ATP and fresh liver tissue homogenate ATP contents were detected by the ATP Assay Kit (Beyotime, Shanghai, China) according to the instructions. The optical density was detected by a multifunctional microplate reader (Infinite 2000 pro, TECAN, Switzerland). The protein content was detected to normalize the ATP level.

### pH measurement of cell culture medium

To detect the pH of the cell culture medium, LO2, HepG2, HepG2.2.15 and Hep3B cells (1 × 10^6^/well) with corresponding transfection or treatment were cultured for 24 h, and the pH of the cell culture medium was measured by a METTLER TOLEDO SevenCompact™ S220 Benchtop pH/Ion Meter (METTLER TOLEDO, Greifensee, Switzerland) according to the instructions.

### Luciferase reporter assay

To examine the promoter activity of NF-κBp65, HepG2 cells (2 × 10^5^/well) were plated in 12-well plates and transfected with the expression plasmid pcDNA3.1-HBx-HA or the vector pcDNA3.1-HA (1000 ng/well) using Lipofectamine 3000 (Invitrogen, Carlsbad, CA, USA). The pGL4.1-NF-κBp65 luciferase reporter plasmid was transfected together with PRL-TK containing the Renilla luciferase plasmid on the next day. For the promoter activity of HK2, HepG2 cells (12-well plate) were transfected with 1000 ng of pCMV-MCS-3flag vector/pCMV-MCS-*NF-κBp65-flag* plasmid or 1000 ng pcDNA3.1-HBx-HA plasmid/50 nM siRNA (negative control or *NF-κBp6*5 siRNA) on the first day and then transfected with 1000 ng of pGL4.1-HK2 luciferase reporter plasmid and PRL-TK plasmid on the next day. The firefly and Renilla luciferase activities were measured 24 h after transfection using a Dual-Luciferase Reporter Assay (E1910, Promega, Fitchburg, WI, USA) with a multifunctional microplate reader (Infinite 2000 pro, TECAN, Switzerland).

### Chromatin immunoprecipitation (ChIP) assay

ChIP assays were performed using a SimpleChIP® Plus Enzymatic Chromatin IP Kit (9005, CST) according to the manufacturer’s instructions. In brief, cells were cross-linked with 1% formaldehyde at room temperature for 10 min. Chromatin was digested using MNase and sonication. The chromatin solution was incubated with ChIP-grade protein G magnetic beads and specific antibodies at 4 °C overnight on a rotating wheel. The ChIP-enriched DNA was purified and subjected to qRT‒PCR analysis using specific primers (Table S4).

### Coimmunoprecipitation (co‑IP)

For the co-IP assay, cells from specific groups were lysed with cell lysis buffer for Western blotting and IP (Beyotime, Shanghai, China). Precleared protein extracts were incubated with anti-HA magnetic beads (1:50, HY-K0201 MCE) or anti-Flag magnetic beads (1:50, HY-K0207 MCE) on a rotator at 4 °C overnight. The protein beads were then rinsed three times with 0.1% PBST, soaked in lysis buffer and heated for 10 min at 70 °C. The supernatant was subjected to western blotting analysis. Whole-cell lysates served as an input control.

### HBV infection

HepG2-NTCP cells were infected by inoculation with HBV-containing human serum from highly viremic patients without antiviral agents and 4% PEG8000 (Beyotime, ST483) at 37 °C overnight. The control group was incubated with healthy volunteers’ serum. Afterwards, the medium containing human serum was removed. HepG2-NTCP cells were washed with PBS 5 times and maintained in complete culture medium (ScienCell, 5201) containing 2% dimethylsulfoxide (Sigma, D2650). Cells and culture medium were collected 5 days after infection and used for follow-up tests. The level of HBV DNA in the culture medium was quantified by the COBAS® TaqMan 48® assay (Roche).

### Statistical analysis

Statistical analysis was conducted using SPSS version 22.0. All data are shown as the means ± standard deviations (SD). Statistical analyses were performed using Student’s t test, one-way ANOVA, repeated-measures ANOVA or the chi-square test. *P* < 0.05 was considered statistically significant.

## Results

### NF-κBp65 was upregulated in hepatitis B virus (HBV)-associated hepatocellular cancer

**Fig. 1:**
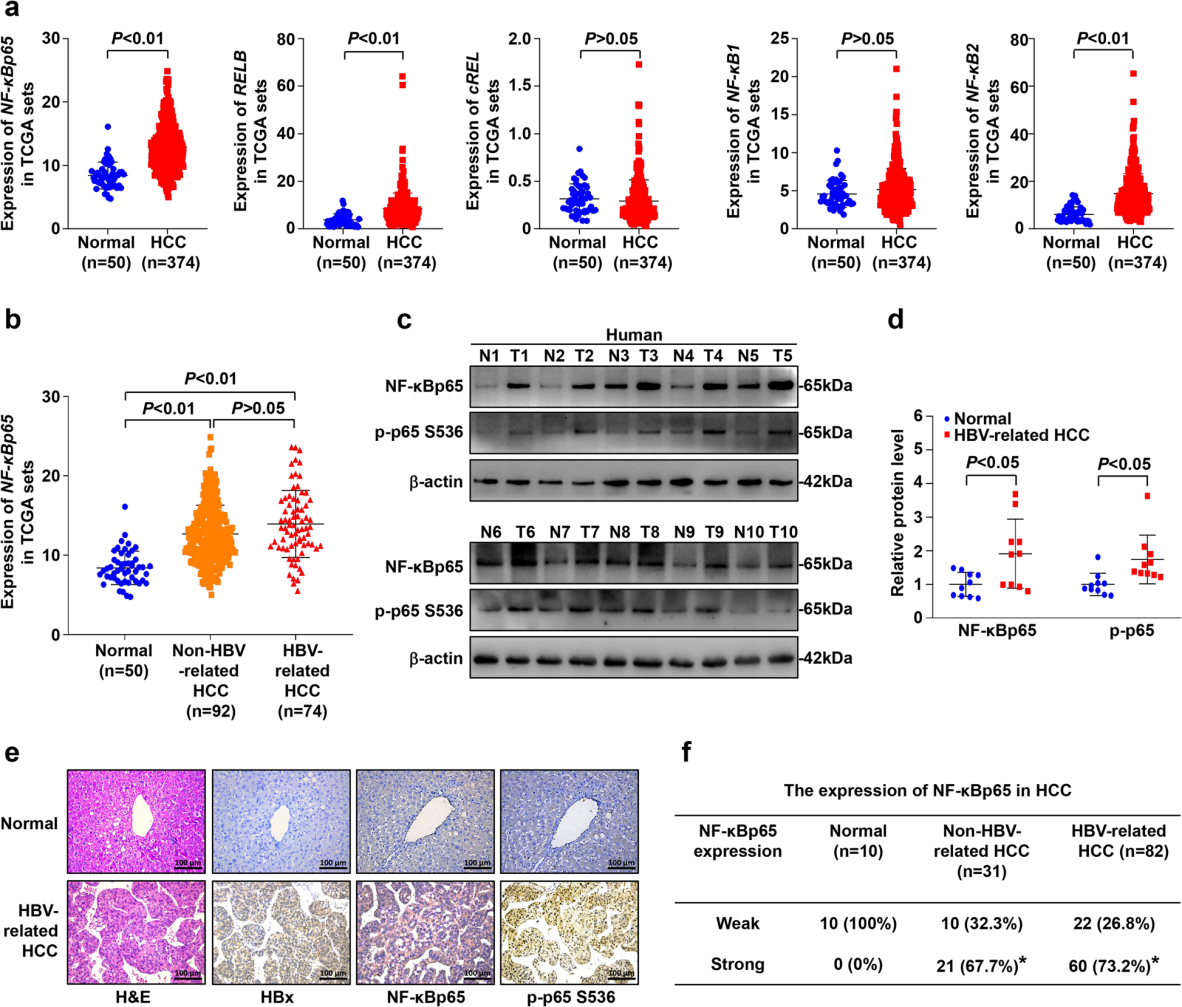
NF-κBp65 was upregulated in hepatitis B virus (HBV)-associated hepatocellular cancer. **a** Expression of NF-κB family members between 374 liver tumour tissues and 50 normal liver tissues in the TCGA profile. **b** Expression of NF-κBp65 in normal liver tissues (*n* = 50), HBV-related HCC (*n* = 74) and non-HBV-related HCC tissues (*n* = 92) in the TCGA profile. **c**, **d** Western blot and densitometry analysis of NF-κBp65 and p-p65 expression in 10 HBV-related HCC liver tissues (T) and in 10 normal controls (N). β-actin was used as the loading control. **e** Representative images of H&E staining, HBx, NF-κBp65 and p-p65 staining of 10 HBV-related HCC samples and 10 normal liver tissue samples. All data are presented as the mean ± SD. *P* < 0.05 using Student’s t test. **f** NF-κBp65 expression was evaluated by IHC in tissue microarrays of human liver cancer tissues in 31 non-HBV-related HCC and 82 HBV-related HCC patients. The expression level was evaluated according to the immunoreactivity score. Chi-square test used. *P < 0.05 compared with normal group

Through analysis of NF-κB subunit expression in published profiles from HCC patients in the TCGA database, we found that NF-κBp65, RelB, and NF-κB2 were upregulated in the HCC samples (374 cases) compared to those in normal liver tissues (50 cases) (*P* < 0.01, Fig. 1a). Moreover, higher NF-κBp65, RelB and NF-κB2 expression was associated with a poor survival rate in HCC patients (Supplementary Fig. S1a-e). To clarify the impact of aetiology between individuals, we further analysed the expression of NF-κBp65 in a total of 50 normal liver tissues, 74 HBV-related HCC patients and 92 non-HBV-related HCC patients in this dataset and found that NF-κBp65 was significantly overexpressed in both HBV-related and non-HBV-related HCC samples compared to that in normal liver tissues (*P* < 0.01) and was slightly upregulated in HBV-related HCC tissues compared to non-HBV-related HCC tissues, although there was no significant difference (*P* > 0.05) (Fig. 1b). Furthermore, to examine the expression of NF-κBp65 in clinical HBV-related HCC specimens from our hospital, ten HBV-related HCC tumour tissues (T) and ten normal liver tissues (N) were analysed. The level of *NF-κBp65* mRNA was higher in HBV-related HCC tissues than in normal liver tissues (Supplementary Fig. S1f). Western blotting (Fig. 1c, d) and immunohistochemistry (Fig. 1e and Supplementary Fig. S1g, h) revealed that NF-κBp65 and phosphorylation of NF-κBp65 (p-p65) were both markedly upregulated in human HBV-related HCC tissues compared to those in normal liver tissues.

We also performed immunohistochemistry (IHC) staining with tissue microarrays of human liver cancer tissues and paired adjacent tissues in 31 non-HBV-related HCC and 82 HBV-related HCC patients (Supplementary Fig. S2a and Table S2). Consistent with the results in the TCGA database, clear NF-κBp65 expression was observed in both non-HBV-related HCC and HBV-related HCC tissues, and strong positive staining of NF-κBp65 was 67.7% in non-HBV-related HCC and 73.2% in HBV-related HCC, however, there was no significant statistical difference (Fig. 1f). Strong NF-κBp65 expression in HCC tissue but not in paracancerous tissue was positively associated with a more advanced tumour size (Supplementary Fig. S2b, c). Moreover, stronger NF-κBp65 expression in tumours had significantly shorter survival than those with weak NF-κBp65 expression in no matter non-HBV-related HCC or HBV-related HCC patients (Supplementary Fig. S2d, e). Taken together, these results strongly indicate that NF-κBp65 is involved in hepatocellular carcinogenesis and is upregulated in HBV-related HCC.

### HBx induced NF-κBp65 expression and enhanced its activation in vitro and in vivo

**Fig. 2:**
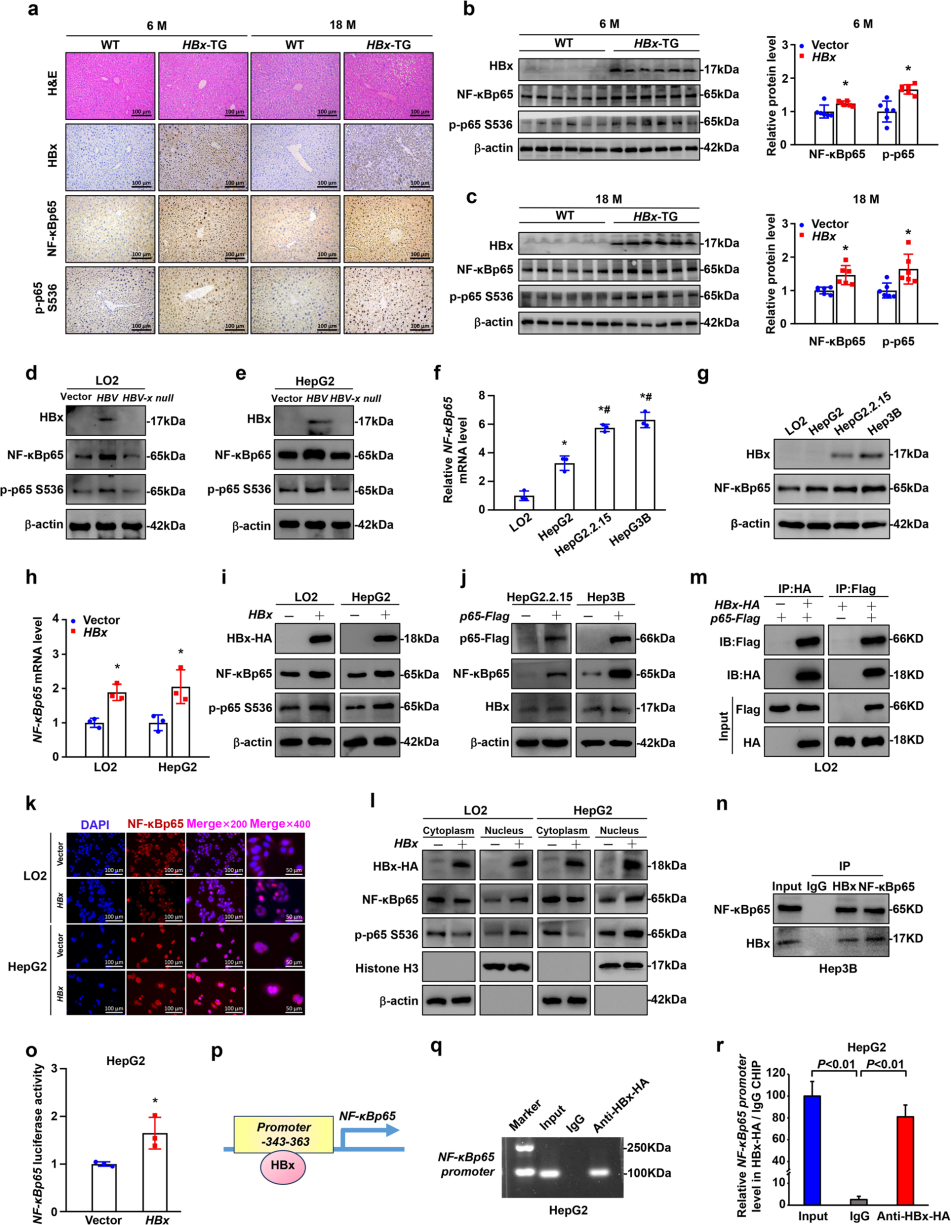
HBx induced NF-κBp65 expression and phosphorylation in vitro and in vivo. **a** Representative H&E staining and HBx, NF-κBp65 and p-p65 staining of liver tissues from WT and HBx-TG mice. Scale bar: 100 μm. *n* = 6 in each group. **b**, **c** Western blot of HBx, NF-κBp65 and p-p65 in liver tissues from WT and HBx-TG mice. *n* = 6 in each group. Quantification is shown on the right graph. **d**, **e** Western blot of HBx, NF-κBp65 and p-p65 protein levels in cells transfected with the indicated plasmids. Relative mRNA levels of *NF-κBp65* **f** and NF-κBp65 protein levels **g** in cell lines. Data are presented as the mean ± SD. *P < 0.05 compared with LO2 cells, ^#^*P* < 0.05 compared with HepG2 cells by one-way ANOVA. **h** Relative *NF-κBp65* mRNA levels in cells transfected with *HA-HBx* plasmid for 24 h. Data are presented as the mean ± SD. *P < 0.05 by Student’s t test. **i** Western blot of HBx-HA, NF-κBp65 and p-p65 in the indicated cells. **j** Western blot of NF-κBp65 and HBx in cells transfected with *Flag-p65* plasmid for 24 h. **k** Immunofluorescence staining of NF-κBp65 in cells. **l** Western blot of HBx, NF-κBp65 and p-p65 in the cytosolic and nuclear fractions. **m** Co-IP between exogenous HBx and NF-κBp65 in LO2 cells transfected with *Flag-p65* and *HA-HBx* plasmids. **n** Co-IP between endogenous HBx and NF-κBp65 in Hep3B cells. **o** Luciferase reporter assay of *NF-κBp65* promoter activity in HepG2 cells transfected with the *HA-HBx* plasmid. Data are presented as the mean ± SD. *P < 0.05 by Student’s t test. **p** Schematic diagram of the promoter region of *NF-κBp65* with a putative HBx binding site. **q**, **r** ChIP‒qPCR and agarose gel to analyse the interaction between HBx and the *NF-κBp65* promoter. Data are presented as the mean ± SD. *P* < 0.05 by one-way ANOVA

To detect the relationship between NF-κBp65 and HBx, we used an *HBx*-transgenic (*HBx*-TG) mouse model with an 86% overall incidence of spontaneous HCC of 11–18 months, while no tumours developed at 6 months [32]. Based on the immunohistochemistry analysis of liver tissues of mice at 6 and 18 months after birth, NF-κBp65 and p-p65 were highly expressed in hepatic tissues of *HBx*-TG mice compared with those of wild-type (WT) mice (Fig. 2a-c and Supplementary Fig. S3a). We then investigated the effect of HBV/HBx on NF-κBp65 expression and activation. We found that NF-κBp65 and p-p65 expression was enhanced in LO2 and HepG2 cells infected with HBV genomic DNA but not HBx-null HBV DNA (Fig. 2d, e and Supplementary Fig. S3b, c), indicating HBx-mediated NF-κBp65 overexpression and activation. Then, we tested NF-κBp65 mRNA and protein expression in the human normal liver cell line LO2, the HCC cell line HepG2, which does not express HBx, and in two HCC cell lines that do express HBx, Hep3B and HepG2.2.15. The results showed increased *NF-κBp65* mRNA and protein levels in HCC cell lines, and the expression of NF-κBp65 was even higher in Hep3B and HepG2.2.15 cells (Fig. 2f, g and Supplementary Fig. S3d). Furthermore, we transfected the *HBx* plasmid into LO2 and HepG2 cells and found that *NF-κBp65* mRNA and protein expressions were significantly upregulated (Fig. 2h, i and Supplementary Fig. S3e). However, HBx protein expression was not upregulated in Hep3B and HepG2.2.15 cells transfected with the *NF-κBp65* plasmid for 48 h (Fig. 2j and Supplementary Fig. S3f). We also found that NF-κBp65 expression and phosphorylation were enhanced in HepG2-NTCP (human hepatoma HepG2 cells expressing sodium taurocholate cotransporting polypeptide) cells infected with HBV virions (Supplementary Fig. S3g-i). To confirm the intracellular distribution of NF-κBp65, immunofluorescence staining of NF-κBp65 was performed. In control LO2 cells, NF-κBp65 was primarily localized in the cytoplasm. However, in LO2-*HBx* cells, NF-κBp65 showed a typical nuclear distribution, indicating HBx-mediated NF-κBp65 activation. A similar status was observed in HepG2-vector and HegG2-*HBx* cells (Fig. 2k and Supplementary Fig. S3j). The nuclear translocation of NF-κBp65 mediated by HBx was further proven by subcellular fractionation showing increased NF-κBp65 and p-p65 in the nucleus of LO2-*HBx* cells compared to those in control cells. Similar results were observed in HepG2-*HBx* cells (Fig. 2l and Supplementary Fig. S3k). Interestingly, from the Western blot analysis of the isolated nuclear and cytosolic fractions, HBx was expressed in both the cytoplasm and nucleus (Fig. 2l).

To investigate the molecular mechanisms involved in regulating NF-κBp65 expression in HBx-related HCC, we performed reciprocal co-IP of Flag-NF-κBp65 and HA-HBx in LO2 and HepG2 cells by transfecting *Flag-NF-κBp65* and *HA-HBx* plasmids into LO2 and HepG2 cells. As shown in Fig. 2m and Supplementary Fig. S3l, co-IP demonstrated that HBx and NF-κBp65 could interact with each other in LO2 and HepG2 cells. Moreover, IP for endogenous proteins also showed that HBx could bind to NF-κBp65 in Hep3B cells (Fig. 2n). Next, we investigated whether HBx affected NF-κBp65 abundance at the transcriptional level in cells. To this end, a dual-luciferase reporter system was used to confirm whether HBx targeted the promoter of *NF-κBp65*. The results indicated that the *NF-κBp65* reporter was activated by HBx (Fig. 2o). Additionally, chromatin immunoprecipitation (ChIP) analysis confirmed that HBx could bind directly to the promoter region of *NF-κBp65* (AGGGAAAACGGGGTAAGGAATC) in HepG2 cells (Fig. 2p-r).

### Hepatocyte-specific *NF-κBp65* deficiency (NF-κBp65^Δhepa^) restrained spontaneous hepatocellular carcinogenesis in *HBx*-TG mice

**Fig. 3:**
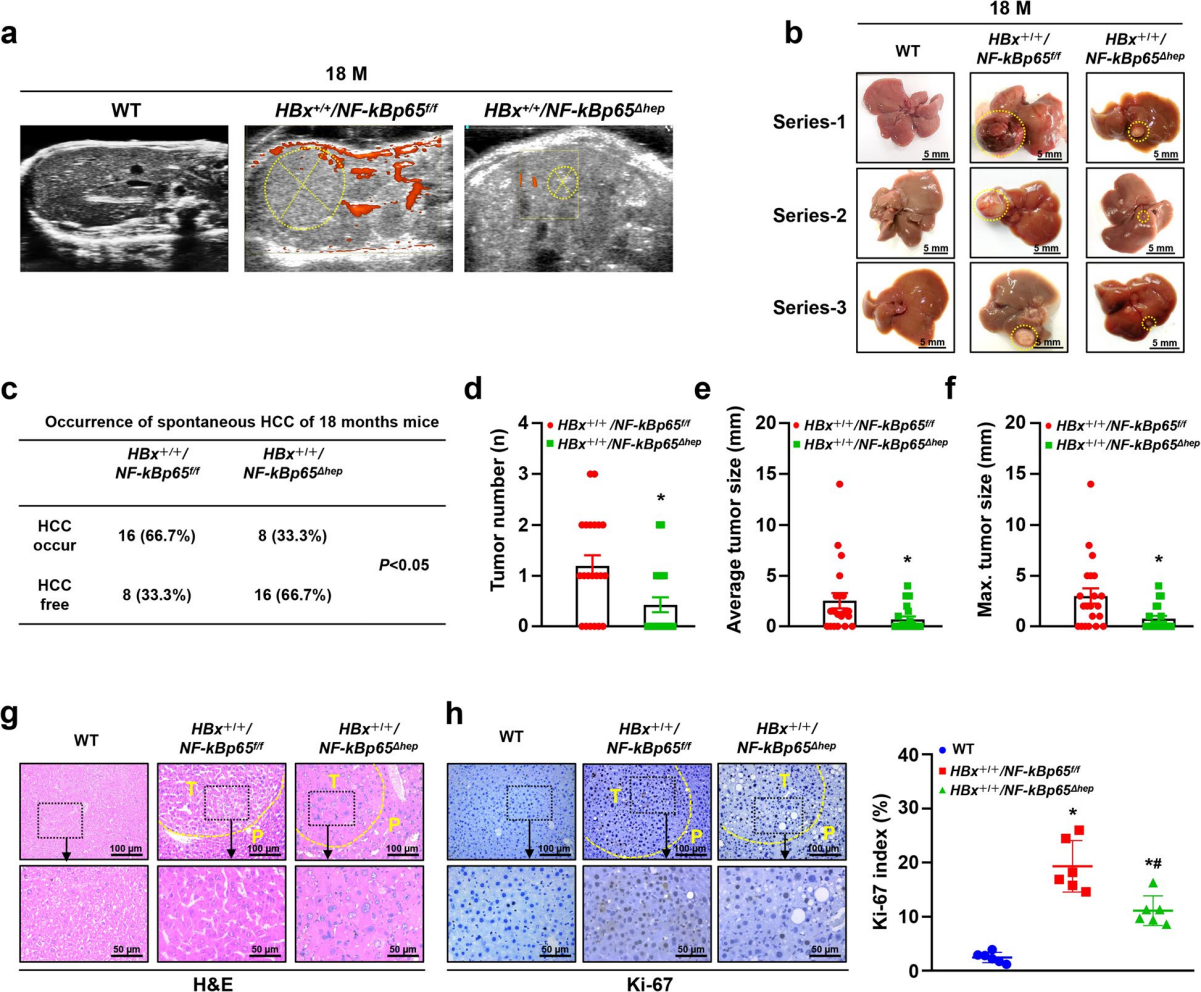
Hepatocyte-specific *NF-κBp65* deficiency (*NF-κBp65*^*Δhepa*^) restrained spontaneous hepatocellular carcinogenesis in *HBx-*TG mice. Representative liver ultrasound images **a** and liver photographs **b** of 18-month-old WT, *HBx*^+*/*+^*/NF-κBp65*^*f/f*^ and *HBx*^+*/*+^*/NF-κBp65*^*Δhep*^ mice. **c** Occurrence of spontaneous HCC in 18-month *HBx*^+*/*+^*/NF-κBp65*^*f/f*^ and *HBx*^+*/*+^*/NF-κBp65*^*Δhep*^ mice, *P* < 0.05 using the chi-square test. Average numbers of liver tumours **d**, average tumour size **e** and maximal tumour size **f** as measured by a calliper in *HBx*^+*/*+^*/NF-κBp65*^*f/f*^ and *HBx*^+*/*+^*/NF-κBp65*^*Δhep*^ mice at 18 months (n = 16 in *HBx*^+*/*+^*/NF-κBp65*^*f/f*^ group, *n* = 8 in *HBx*^+*/*+^*/NF-κBp65*^*Δhep*^ group). Data are presented as the mean ± SD. *P < 0.05 by Student’s t test. Representative H&E staining **g** and Ki-67 staining **h** of 18-month-old WT liver tissue and spontaneous liver tumours in *HBx*^+*/*+^*/NF-κBp65*^*f/f*^ and *HBx*^+*/*+^*/NF-κBp65*^*Δhep*^ mice. The Ki-67 index was scored. Data are expressed as the mean ± SD (*n* = 6 in each group), *P < 0.05 compared with WT mice, ^#^*P* < 0.05 compared with *HBx*^+*/*+^*/NF-κBp65*^*f/f*^ mice by one-way ANOVA

To further assess the role of NF-κBp65 in HBV-related hepatocellular carcinogenesis, we established a hepatocyte-specific deletion of the *NF-κBp65* gene in *HBx*-TG mice by crossing *HBx*-TG (*HBx*^+*/*+^*/NF-κBp65 *^*f/f*^) with hepatocyte *NF-κBp65*^−/−^ littermates. Livers of male *HBx*^+/+^*/NF-κBp65 *^*f/f*^ and *HBx*^+*/*+^*/NF-κBp65*^*Δhepa*^ mice were harvested at 6 months (*n* = 6 in each group), 12 months (*n *= 6 in each group) and 18 months (*n* = 24 in each group). Ultrasound examination was performed, and tumour incidence, tumour weight and tumour size were evaluated at 18 months. Distinct hepatocellular carcinoma developed spontaneously in 16 male *HBx*^+/+^*/NF-κBp65 *^*f/f*^ mice (16/24) and 8 male *HBx*^+/+^/*NF-κBp65*^*Δhepa*^ mice (8/24) at 18 months, indicating that the hepatocellular carcinoma incidence was significantly reduced in *HBx*^+/+^/*NF-κBp65*^*Δhepa*^ mice compared with *HBx*^+/+^/*NF-κBp65 *^*f/f*^ mice (Fig. 3a-c). As shown in Fig. 3d-f, the tumour number, average tumour size and maximum tumour size were also notably decreased in *HBx*^+*/*+^/*NF-κBp65*^*Δhepa*^ mice compared to those in *HBx*^+*/*+^/*NF-κBp65*^*f/f*^ mice. Histopathological staining was used to confirm mouse hepatocellular cancer (Fig. 3g). Ki-67 staining was performed to analyse the proliferation of liver tumours, showing that the number of Ki-67-positive cells was reduced in *HBx*^+*/*+^/*NF-κBp65*^*Δhepa*^ mice compared to that in *HBx*^+*/*+^/*NF-κBp65 *^*f/f*^ mice (Fig. 3h). These results demonstrated that hepatocyte-specific *NF-κBp65* deficiency restrains spontaneous hepatocarcinogenesis by downregulating hepatic proliferation in *HBx*-TG mice.

### HBx enhanced aerobic glycolysis in hepatocellular carcinogenesis

**Fig. 4:**
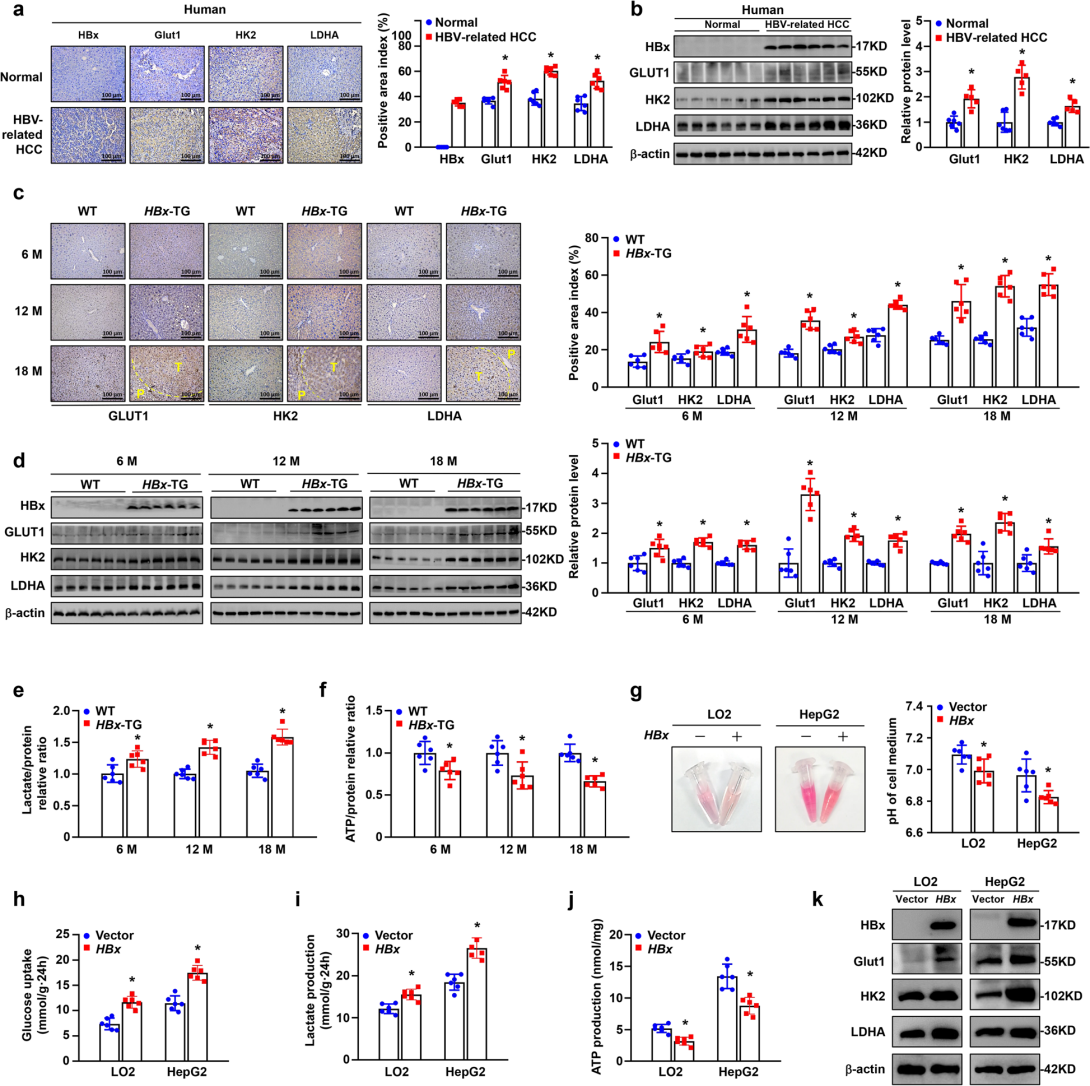
HBx activated aerobic glycolysis in hepatocellular carcinogenesis. **a** Representative images and quantification of HBx, Glut1, HK2 and LDHA staining in human normal liver tissues and in HBV-related HCC tissues. Scale bar, 100 μm. *n* = 6 in each group. Quantification of IHC is shown in the right graph. **b** Western blot analysis of HBx, Glut1, HK2 and LDHA protein expression in human normal liver tissues and HCC tissues. β-actin was used as the loading control. Quantification of proteins is shown in the right graph. *n* = 6 in each group. **c** Representative images and quantification of Glut1, HK2 and LDHA staining in liver tissues from WT and *HBx*-TG mice at 6, 12 and 18 months. Scale bar: 100 μm. *n* = 6 in each group. **d** Whole liver homogenates were analysed by Western blot using the indicated antibodies. Quantification of proteins is shown in the right graph. *n* = 6 in each group. Lactate **e** and ATP content **f** were detected in the WT and *HBx*-TG mice liver tissues at 6, 12 and 18 months. *n* = 6 in each group. **g** Representative images of the colour change of the corresponding culture medium after 24 h of cell culture. pH of cell culture medium is shown in the right graph. Glucose uptake **h**, lactate secretion **i**, and intracellular ATP content **j** were measured in corresponding cells after 24 h of cell culture. **k** Cell lysates were analysed by Western blot using the indicated antibodies. All data are expressed as the mean ± SD. *P < 0.05 by Student’s t test

Aerobic glycolysis, also termed the Warburg effect, is a significant hallmark of cancer [13]. Previous studies have reported that HBx is involved in aerobic glycolysis [8–11]. In this study, we tested the content of lactic acid in human HBV-related HCC tissues and normal liver tissues (*n* = 6 in each group). The results showed that lactate was much higher in HBV-related HCC tissues than in normal liver tissues (Supplementary Fig. S4a). Then, we performed IHC and WB of the key glycolysis proteins GLUT1, HK2 and LDHA in the above specimens. The expressions of GLUT1, HK2 and LDHA were significantly higher in human HBV-related HCC samples than in normal liver tissues (Fig. 4a, b). Higher GLUT1, HK2 and LDHA expressions were also shown in liver tissues of 6-, 12-, and 18-month-old *HBx*-TG mice compared to those of corresponding WT mice according to IHC and WB analysis (Fig. 4c, d). Moreover, the lactate content of fresh liver homogenate was significantly increased in the liver tissues of 6-, 12-, and 18-month-old *HBx*-TG mice compared to that of corresponding WT mice (Fig. 4e). The ATP content of fresh liver homogenate was significantly decreased in the liver tissues of 6-, 12-, and 18-month-old *HBx*-TG mice compared to that of corresponding WT mice (Fig. 4f). Consistently, *HBx* stably transfected into LO2 and HepG2 cells led to a more acidic environment, as indicated by the colour and pH of the medium (Fig. 4g), increased glucose uptake and lactate production and decreased ATP production compared to the vector cells (Fig. 4h-j). Furthermore, Western blotting showed that GLUT1, HK2 and LDHA increased in LO2-*HBx* and HepG2-*HBx* cells compared to the vector cells (Fig. 4k and Supplementary Fig. S4b, c). Taken together, these findings indicate that HBx facilitates aerobic glycolysis in hepatocellular carcinogenesis.

### Inhibition of NF-κBp65 downregulated HBx-induced aerobic glycolysis

**Fig. 5:**
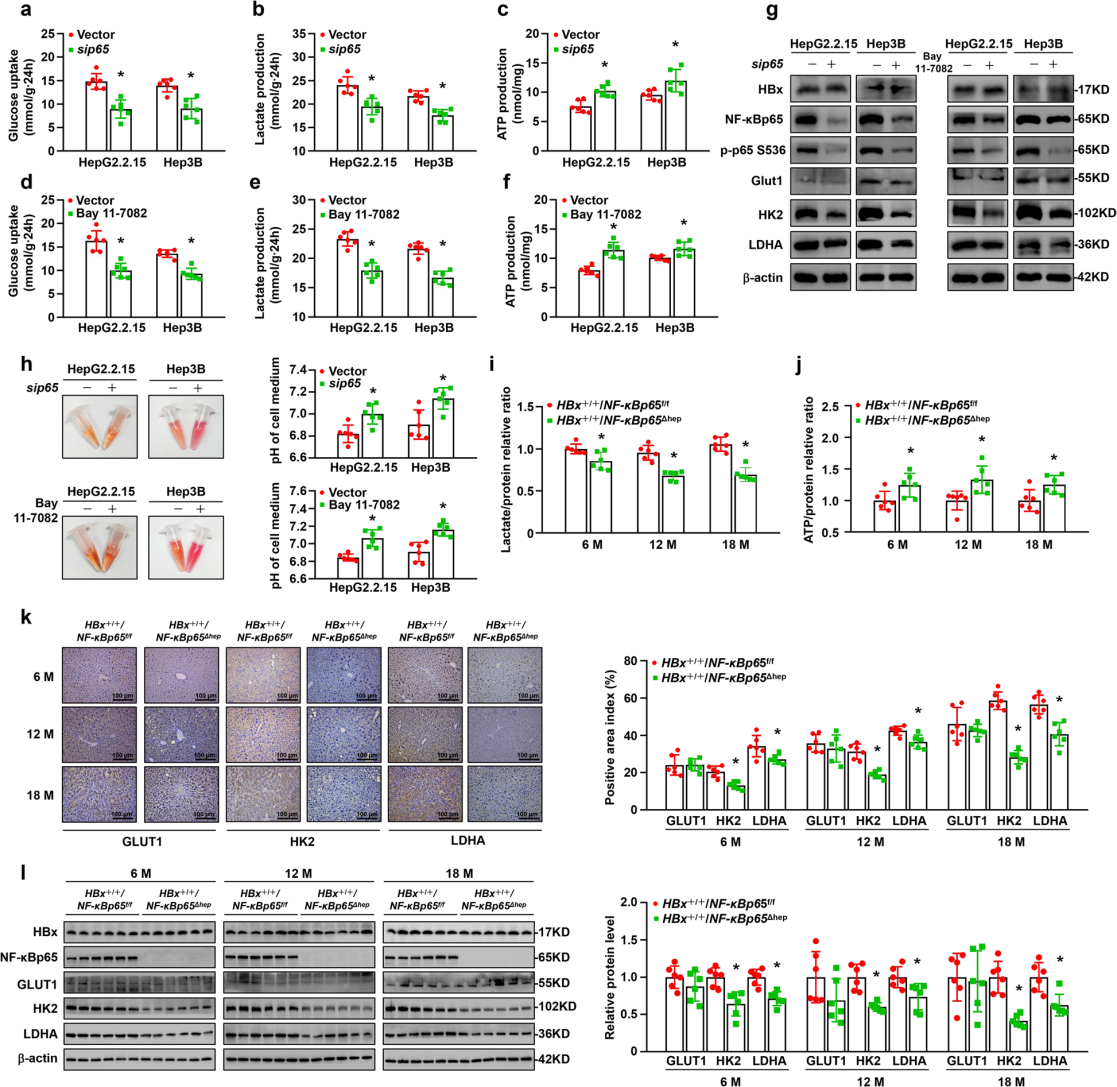
Inhibition of NF-κBp65 downregulated HBx-induced aerobic glycolysis. Glucose uptake **a**, **d**, lactate production **b**, **e**, and ATP production **c**, **f** were detected in different groups of HepG2.2.15 and Hep3B cells. **g** Cell lysates were analysed by Western blot using the indicated antibodies. **h** Representative images of the colour change of the corresponding culture medium after 24 h of cell culture. pH of cell culture medium is shown in the right graph. Lactate **i** and ATP content **j** were detected in the *HBx*^+*/*+^*/NF-κBp65*^*f/f*^ and *HBx*^+*/*+^*/NF-κBp65*^*Δhep*^ mice liver homogenates. *n* = 6 in each group. **k** Representative images and quantification of Glut1, HK2 and LDHA staining in *HBx*^+*/*+^*/NF-κBp65*^*f/f*^ and *HBx*^+*/*+^*/NF-κBp65*^*Δhep*^ mice liver tissues. *n* = 6 in each group. **l** Whole mice liver homogenates were analysed by Western blot using the indicated antibodies. Quantification of proteins is shown in the right graph. *n* = 6 in each group. All values are the mean ± SD. *P < 0.05 by Student's t test

The preceding data demonstrated that HBx promoted the expression and activation of NF-κBp65 and induced glycolysis in HCC. To further explore the effects of NF-κBp65 on the glucose metabolism induced by HBx, we knocked down *NF-κBp65* by small interfering RNA (si*NF-κBp65*) in HepG2.2.15 and Hep3B cells. Decreased glucose uptake, diminished lactate production and enhanced ATP production were found in the *NF-κBp65* knockdown group (Fig. 5a-c). To assess whether inhibition of the NF-κB canonical pathway was associated with concomitant suppression of glycolysis in HCC, HepG2.2.15 and Hep3B cells were treated with Bay 11–7082 (an inhibitor of NF-κB). The NF-κB inhibitor also decreased glucose uptake and lactate production and increased ATP production in HepG2.2.15 and Hep3B cells (Fig. 5d-f). Western blot analysis showed a decrease in NF-κBp65, p-p65, HK2 and LDHA in *NF-κBp65* knockdown cells and inhibition of NF-κB cells compared to those in control cells (Fig. 5g). Consistently, HepG2.2.15 and Hep3B cells transfected with si*NF-κBp65* or treated with Bay 11–7082 led to a less acidic environment, as indicated by the colour and pH of the medium (Fig. 5h). Moreover, the lactate content of fresh liver homogenate was significantly decreased in the liver tissues of 6-, 12-, and 18-month-old *HBx*^+*/*+^*/NF-κBp65*^Δhepa^ mice compared to that of corresponding *HBx*^+/+^/*NF-κBp65*^*f/f*^ mice (Fig. 5i). The ATP content of fresh liver homogenate was significantly increased in the liver tissues of 6-, 12-, and 18-month-old *HBx*^+*/*+^*/NF-κBp65*^*Δhepa*^ mice compared to that of *HBx*^+*/*+^*/NF-κBp65*^*f/f*^ mice at the same age (Fig. 5j). Immunohistochemistry and Western blot analysis demonstrated a significant decrease in the expression of glycolytic proteins (particularly HK2) in hepatocyte-specific *NF-κBp65* knockout tissues compared to those in control tissues at the corresponding age (Fig. 5 k, l). These data reveal that inhibition of NF-κBp65 decreases the glycolysis that is triggered by HBx.

### Inhibition of NF-κBp65 suppressed HBx-induced proliferation by downregulating aerobic glycolysis

**Fig. 6:**
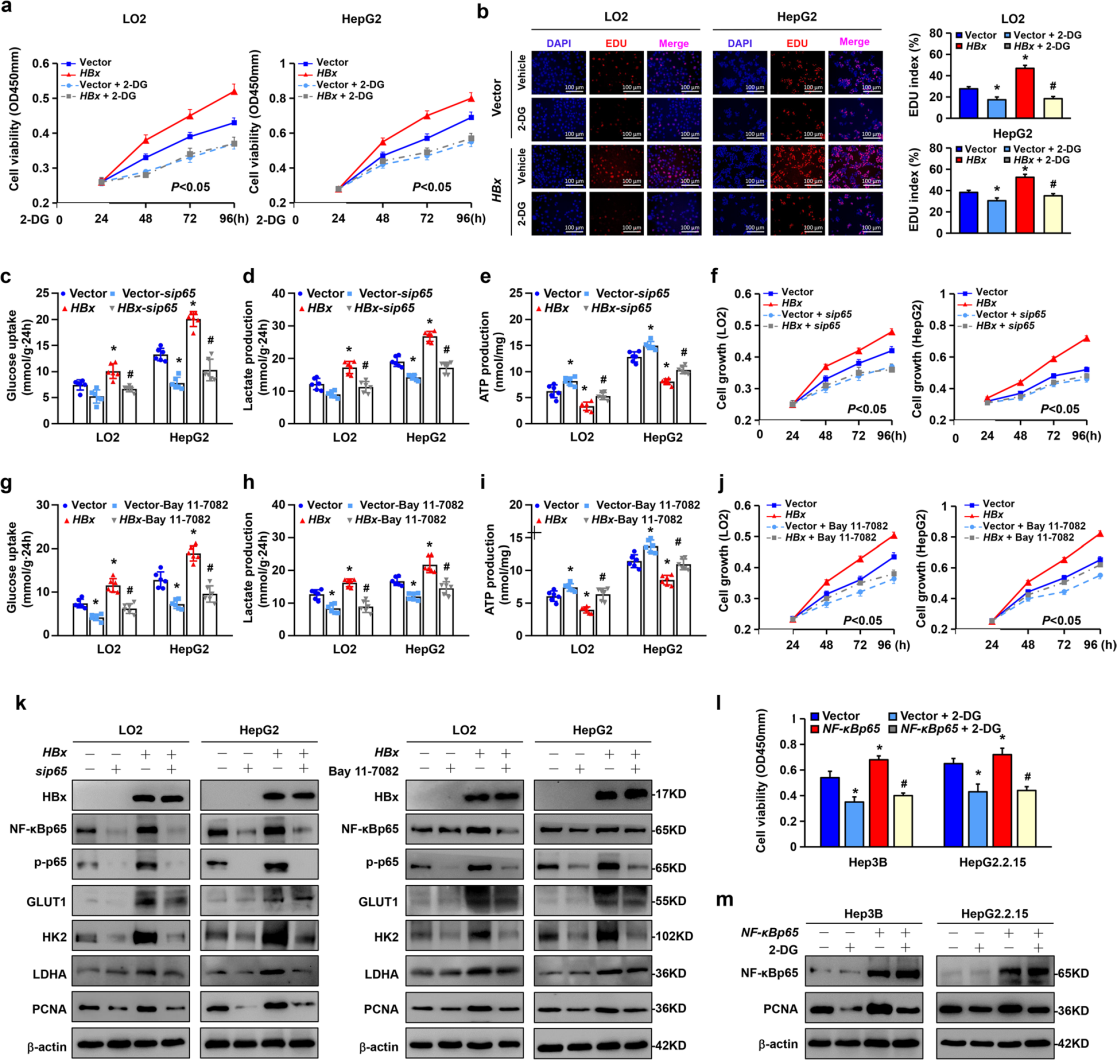
Inhibition of NF-κBp65 suppressed HBx-induced proliferation by downregulating aerobic glycolysis. **a** CCK-8 assay to test cell viability in the indicated cells treated with or without 2-DG. **b** LO2 and HepG2 cells with or without *HA-HBx* expression were treated with 2-DG (10 mM) for 24 h. An EdU incorporation assay was used to examine cell proliferation. Scale bar: 100 μm. The EdU index was quantified. LO2 and HepG2 cells with or without *HA-HBx* expression were transiently transfected with *NF-κBp65* small interfering RNA or treated with Bay 11–7082 (10 µM) for 24 h. Glucose uptake **c**, **g**, lactate production **d**, **h**, and ATP content **e**, **i** were detected in cells. **f**, **j** Cell growth of different groups was detected by CCK-8 assay. **k** Cell lysates were analysed by Western blot using the indicated antibodies. **l** Hep3B and HepG2.2.15 cells were transiently transfected with *NF-κBp65* plasmids, followed by treatment with 2-DG (10 mM) for 24 h. Cell viability was examined by CCK-8 assay. **m** Western blot analysis of NF-κBp65 and PCNA protein expression in the above cells. β-actin was used as the loading control. The experiment was repeated three times. All values are the mean ± SD. *P* < 0.05 by repeated-measures ANOVA or *P < 0.05 compared to the vector group, ^***#***^*P* < 0.05 compared to the indicated group by one-way ANOVA

NF-κBp65 plays a vital role in glucose metabolic reprogramming, and it has been associated with proliferation and survival in previous studies [24–26]. To investigate the interplay between glycolysis and proliferation mediated by NF-κBp65 in HBx-related cells, we used 2-deoxy-D-glucose (2-DG), which was used to inhibit the transformation of glucose-6-phosphate from glucose to suppress glycolytic metabolism. We first used the CCK-8 assay to detect the relationship between HBx-induced glycolysis and proliferation. Compared to the vector cells, the LO2-*HBx* and HepG2-*HBx* cells were more susceptible to blockage of glycolysis (Fig. 6a). Moreover, EdU staining showed that overexpression of *HBx* promoted the proliferation of LO2 and HepG2 cells. Treatment with 2-DG attenuated HBx-induced proliferation (Fig. 6b). Consistently, RT‒qPCR and Western blotting showed that HBx upregulated the expression of the proliferation-related gene PCNA at the mRNA and protein levels, but this effect was reversed after treatment with 2-DG (Supplementary Fig. S5a-d). Taken together, these results revealed that glycolytic metabolism plays a pivotal role in maintaining cell proliferation induced by HBx. Further experiments were conducted with interference of *NF-κBp65* or an NF-κB inhibitor (Bay 11–7082) in LO2-*HBx* and HepG2-*HBx* cells. The levels of glucose uptake and lactate production were downregulated with si*NF-κBp65* treatment and accompanied by increased ATP production in LO2-*HBx* and HepG2-*HBx* cells (Fig. 6c-e). Cell viability was inhibited with the interference of *NF-κBp65* in LO2-*HBx* and HepG2-*HBx* cells (Fig. 6f). Treatment with Bay 11–7082 also showed consistent results in glucose, lactate and ATP biochemical tests (Fig. 6g-i). Cell growth was also slowed by treatment with the NF-κB inhibitor in LO2-*HBx* and HepG2-*HBx* cells (Fig. 6j). Then, we performed western blot analysis to detect the variation in glycolytic enzymes and PCNA in the corresponding treatment. Knockdown of *NF-κBp65* or deactivation of the canonical NF-κB pathway markedly decreased the expression of the glycolytic rate-limiting enzymes HK2 and PCNA (Fig. 6k). Next, we treated Hep3B and HepG2.2.15 cells with 2-DG after transfection with the *NF-κBp65* plasmid. The results of CCK-8 and Western blot analyses showed that NF-κBp65 promoted cell viability and PCNA expression. When glycolysis was blocked, cell growth was significantly downregulated, and PCNA was reduced (Fig. 6l, m). These results indicate that inhibition of glycolysis results in NF-κBp65-mediated proliferation reduction. Altogether, NF-κBp65-mediated aerobic glycolysis played an important role in maintaining HBx-induced hepatocellular proliferation.

### HBx induced aerobic glycolysis via the NF-κBp65/HK2 pathway to reprogram glycolytic metabolism in hepatocellular carcinogenesis

**Fig. 7:**
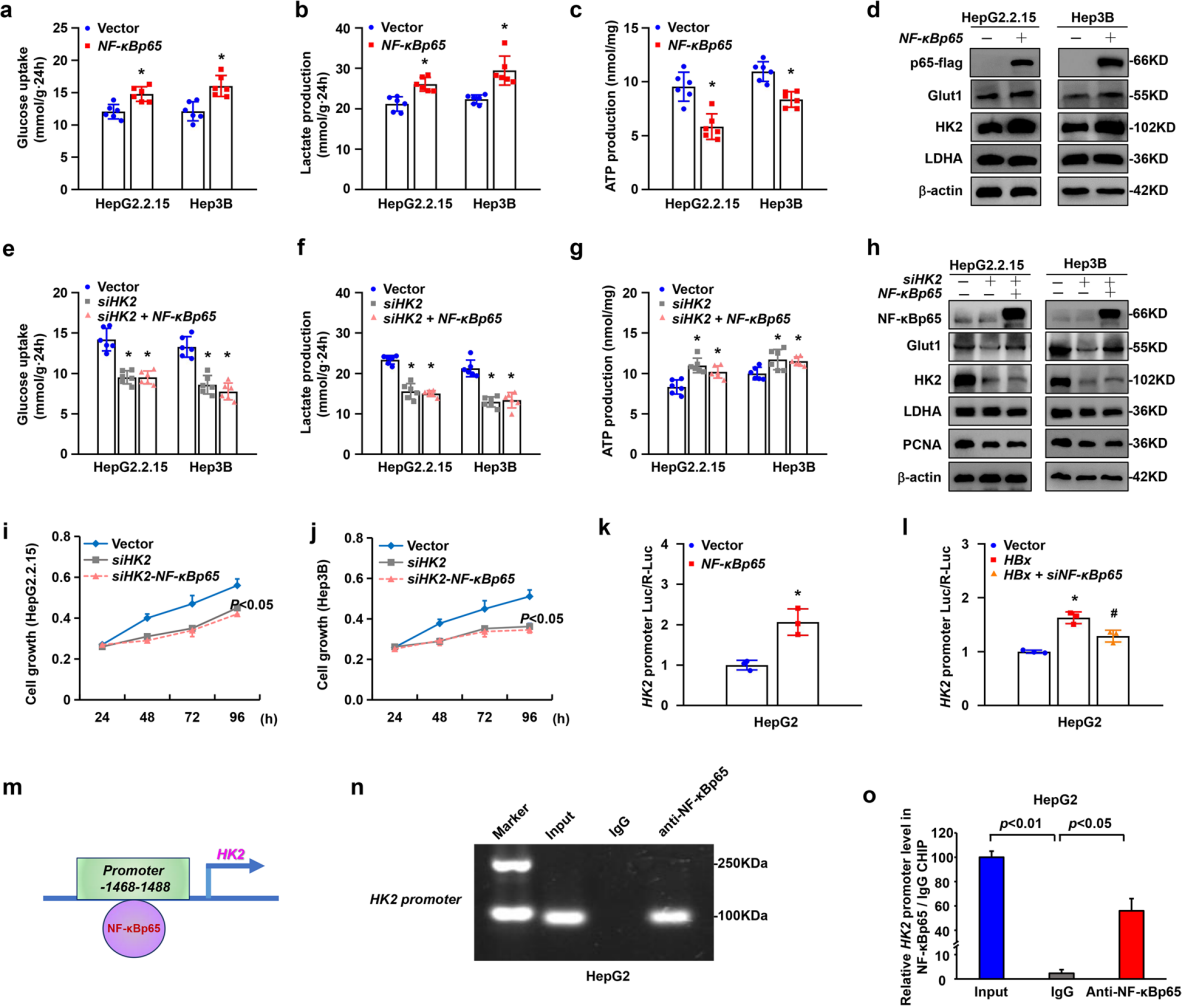
HBx induced aerobic glycolysis via the NF-κBp65/HK2 pathway to reprogram glycolytic metabolism in hepatocellular carcinogenesis. Glucose uptake **a**, lactate production **b**, and ATP production **c** were detected in HepG2.2.15 and Hep3B cells with or without transient transfection with *NF-κBp65* plasmids. Data are presented as the mean ± SD. *P < 0.05 by Student’s t test. **d** Cell lysates were analysed by Western blot using the indicated antibodies. Glucose uptake **e**, lactate production **f**, and ATP production **g** were detected in HepG2.2.15 and Hep3B cells transfected with *HK2* siRNA or combined with the *NF-κBp65* plasmid. Data are presented as the mean ± SD. *P < 0.05 compared to the vector cells by one-way ANOVA. **h** Cell lysates were analysed by Western blot using the indicated antibodies. **i**, **j** Cell growth of the above cells was detected by CCK-8 assays. Data are presented as the mean ± SD. *P* < 0.05 by repeated-measures ANOVA. **k** Luciferase reporter assay of *HK2* promoter activity in HepG2 cells transfected with the *NF-κBp65* plasmid. Data are presented as the mean ± SD. *P < 0.05 by Student’s t test. **l** Luciferase reporter assay of *HK2* promoter activity in HepG2 cells transfected with *HBx* plasmid and *NF-κBp65* siRNA. Data are presented as the mean ± SD. *P < 0.05 compared to the vector cells and ^***#***^*P* < 0.05 compared to the *HBx* cells by one-way ANOVA. **m** Schematic diagram of the promoter region of *HK2* with a putative NF-κBp65 binding site. **n**, **o** ChIP‒qPCR assays and agarose gel were performed to analyse the interaction between NF-κBp65 and the *HK2* promoter. Data are presented as the mean ± SD. *P* < 0.05 by one-way ANOVA

To investigate the mechanism of glycolytic metabolism mediated by NF-κBp65 in HBx-expressing cells, we overexpressed *NF-κBp65* in HepG2.2.15 and Hep3B cells with plasmids. It led to upregulated cell aerobic glycolysis with higher glucose uptake and lactate production and less ATP production in the experimental groups compared to that in the control groups (Fig. 7a-c). Cells with *NF-κBp65* also showed increased expression of glycolytic proteins, especially HK2, as shown by western blotting (Fig. 7d and Supplementary Fig. S6a, b). Then, interference with *HK2* in HepG2.2.15 and Hep3B cells resulted in downregulation of glycolysis, which showed decreased glucose uptake and lactate production and augmented ATP production (Fig. 7e-g). The expression of PCNA was reduced in *HK2*-silenced cells, as shown by western blot analysis (Fig. 7h and Supplementary Fig. S6c, d). However, overexpression of *NF-κBp65* did not reverse the reduction in glycolysis induced by *HK2* silencing (Fig. 7e-h). Proliferation was assessed by CCK-8 proliferation curve analysis. Loss of *HK2* in HepG2.2.15 and Hep3B cells also led to reduced cell proliferation, and overexpression of *NF-κBp65* could not reverse the retardation of proliferation (Fig. 7i, j). Furthermore, RT‒qPCR showed that *HK2* mRNA was significantly increased in HepG2.2.15 and Hep3B cells overexpressing *NF-κBp65* but decreased in *NF-κBp65-*silenced cells compared to vector-transfected cells (Supplementary Fig. S6e, f). These results suggested that HK2 was a downstream target of NF-κBp65 and might be regulated by NF-κBp65 at the transcriptional level. Therefore, we performed luciferase reporter assays to investigate the mechanism between NF-κBp65 and HK2. The results revealed that the *HK2* reporter was activated by NF-κBp65 (Fig. 7k). *HK2* promoter activity was upregulated by overexpression of *HBx* in HepG2 cells, while this upregulation could be reversed by silencing *NF-κBp65* in HepG2-*HBx* cells (Fig. 7l). Interestingly, NF-κBp65 was found to bind directly to the *HK2* promoter (GCCTTGCCTCAATTTCCTCATC) according to the ChIP assay (Fig. 7m-o).

### Lactate, but not pyruvate, promoted proliferation via PI3K/Akt signalling in hepatocellular carcinogenesis

**Fig. 8:**
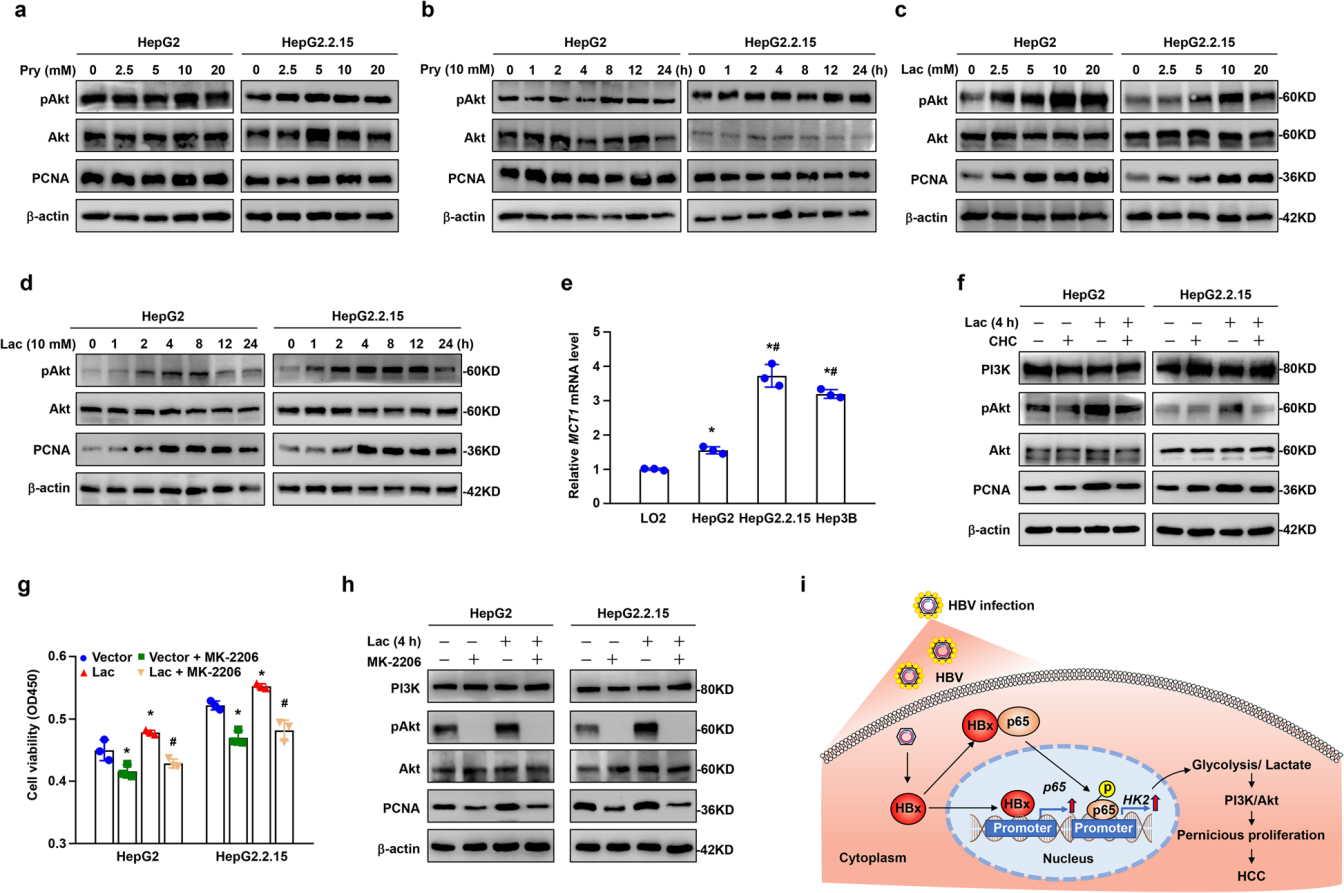
Lactate, but not pyruvate, promoted proliferation via PI3K/Akt signalling in hepatocellular carcinogenesis. Western blot analysis of p-Akt, Akt and PCNA protein expression at indicated doses of sodium pyruvate (Pry) for 4 h **a** or at different time points with 10 mM Pry **b**, at indicated doses of lactate (Lac) for 4 h **c** or at different time points with 10 mM Lac **d** in HepG2 and HepG2.2.15 cells. β-actin was used as the loading control. **e** *MCT1* mRNA levels were measured in different cells by RT‒qPCR. Data are presented as the mean ± SD. One-way ANOVA was used to calculate *P* values. *P < 0.05 versus LO2 cells, ^***#***^*P* < 0.05 versus HepG2 cells. **f** HepG2 and HepG2.2.15 cells were treated with or without 10 mM lactate for 4 h, and CHC (α-cyano-4-hydroxycinnamate, MCT1 transporter inhibitor, 5 mM) was added to the cells 30 min before lactate treatment. PI3K, p-Akt, Akt, and PCNA protein expression was analysed by Western blotting. **g** HepG2 and HepG2.2.15 cells were treated with or without 10 mM lactate for 4 h, and MK-2206 (Akt inhibitor, 10 µM) was added to the cells 30 min before lactate treatment. Cell viability was examined by CCK-8 assay. All data are presented as the mean ± SD. One-way ANOVA was used to calculate *P* values. *P < 0.05 versus vector groups, ^***#***^*P* < 0.05 versus lactate treatment groups. **h** Western blot analysis of PI3K, p-Akt, Akt, and PCNA protein expression in HepG2 and HepG2.2.15 cells with the above treatments. **i** Schematic diagram

Tumour cell metabolism is dominated by the Warburg effect, where tumour cells produce large amounts of lactate from pyruvate under aerobic conditions. Lactate is secreted into the tumour microenvironment and provides an acidic environment to exacerbate malignant tumour properties [12, 13, 34, 35]. Our previous reports showed that prominent activation of the PI3K/Akt pathway was essential for proliferation in HCC [29]. We considered whether lactate and pyruvate could facilitate the PI3K/Akt pathway to promote the proliferation of HCC cells. First, we treated the HCC cell lines HepG2 and HepG2.2.15 with pyruvate and lactate, respectively. We observed that lactate, but not pyruvate, significantly induced Akt phosphorylation (pAkt) and PCNA expression (Fig. 8 a-d). The maximal response of pAkt induced by lactate was observed at a concentration of 10 mM by 4 h. Consequently, this dose and time point was used in all subsequent assays. The transport of lactate across the cell membrane is mainly exerted by transporters of the monocarboxylate transporter (MCT) family [36]. Of these, MCT1 is typically involved in the import of lactate, while MCT4 is adapted for the export of lactic acid from glycolytic cells. Then, we found that the expression of *MCT1* mRNA was much higher in HCC cell lines than in the immortal human liver cell line LO2 (Fig. 8e). This indicates that lactic acid is more readily absorbed in liver tumour cells. Furthermore, we observed that the MCT1 inhibitor α-cyano-4-hydroxycinnamate (CHC) antagonized lactate-dependent activation of Akt and subsequently PCNA expression in HCC cells (Fig. 8f). Next, we treated HepG2 and HepG2.2.15 cell lines with MK-2206 (Akt inhibitor) after lactate treatment and found that cell viability was significantly downregulated by blockage of lactate-induced Akt phosphorylation (Fig. 8g). Western blotting analysis also showed that the Akt inhibitor antagonized lactate-induced activation of Akt and subsequent PCNA expression in HCC cells (Fig. 8h). Therefore, blocking the lactate/MCT1/PI3K/Akt pathway suppressed the malignant proliferation features of HCC cells.

## Discussion

Increasing studies have suggested that HBx is a multifunctional regulatory factor and plays a critical role in the initiation of HCC [4–7]. In the present study, we showed that HBx overexpression upregulated the NF-κBp65/HK2 axis and enhanced aerobic glycolysis and cell proliferation. By using newly established *HBx*^+*/*+^*/NF-κBp65*^+*/*+^ and *HBx*^+*/*+^*/NF-κBp65*^*Δhepa*^ spontaneous HCC formation transgenic mouse models, we present convincing experimental evidence that hepatocyte-specific *NF-κBp65* deficiency restrained spontaneous HCC formation in *HBx*-TG mice. We also found that inhibition of the canonical NF-κB pathway suppressed the NF-κBp65/HK2 axis and delayed cell proliferation. Moreover, overproduced lactate in turn facilitated a more malignant phenotype of HCC cells via PI3K/Akt signalling. These findings confirmed the crucial role of these alterations and subsequent aberrant glycolysis in tumour progression during HCC.

NF-κB, a collection of transcription factors, regulates gene expression in a diverse spectrum of biological processes, including inflammation, immunity, differentiation, proliferation, as well as the metabolic state of cells [18, 21, 22]. In most resting cells, NF-κB dimers are sequestered in the cytoplasm by inhibitor of κB (IκB) family. The canonical NF-κB activation is mainly in response to the stimuli such as proinflammatory cytokines as well as bacterial and viral antigens. This activation eventually causes the nuclear accumulation of NF-κB dimers to promote gene expression. And NF-κBp65-p50 is the most abundant transcriptionally active heterodimer [37]. Furthermore, NF-κB subunits also contain sites for phosphorylation and other post-translational modifications which are important for activation [17]. In this study, we found that HBx could upregulate the expression of NF-κBp65 in a transcriptional manner. In the nucleus, HBx could bind directly to the promoter of *NF-κBp65* and increase the expression of NF-κBp65. Our previous report revealed that phosphorylation of NF-κBp65 plays a vital role in hepatocellular carcinogenesis [24]. Therefore, we hypothesized whether HBx could induce the phosphorylation of NF-κBp65. In our study, we found that HBx overexpression promoted the translocation of NF-κBp65 from cytoplasm to the nucleus and increased the level of p-p65, thus p-p65 could bind to the promoter of glycolytic gene and activates gene expression. Interestingly, we also found that HBx could directly bind to NF-κBp65 protein. Although previous study has illustrated that HBx increases the interleukin-1β (IL-1β)-induced NF-κB activation via interaction with evolutionarily conserved signalling intermediate in Toll pathways (ECSIT) [38], however, further studies are still needed to explore other molecular mechanisms of how HBx inducing phosphorylation of NF-κBp65.

Aerobic glycolysis, also referred to as the Warburg effect, is an important feature distinguishing cancer cells from healthy cells and is characterized by upregulated glucose consumption and lactate production even under normoxic conditions [12]. NF-κB activity controls the balance between glycolysis and mitochondrial respiration. However, the role of glucose metabolic regulation of NF-κB is controversial in diverse situations. In mouse embryonic fibroblasts (MEFs), knockdown of *NF-κBp65* resulted in increased expression of GLUT3. NF-κB facilitates mitochondrial respiration and restricts glycolysis [39]. In contrast, an earlier report revealed that enhanced activation of NF-κB increased glucose uptake and glycolysis in p53^−/−^ MEFs via upregulation of GLUT3 [22]. These differences may be due to a confounding factor of different p53 statuses and may need further exploration in animal models. Nevertheless, the proglycolytic role of NF-κBp65 in some studies of different cancer cells has shown consistent findings, e.g., inhibition of the classical NF-κB pathway results in downregulation of glycolytic enzymes, particularly hexokinase 2 (HK2), and is subsequently accompanied by decreased glycolysis in sarcoma cells [25] and primary central nervous system lymphoma [26]. This is consistent with our findings that inhibition of NF-κBp65 resulted in a dramatic decrease in glycolysis in HBx-expressing cells and in *HBx*-TG mice. Here, we underscore the pivotal regulatory role of the canonical NF-κB pathway in promoting aerobic glycolysis and subsequent proliferation during tumour progression in HBx-initiated HCC.

Hexokinase (HK) is the first rate-limiting enzyme in glucose metabolism and can catalyse glucose into glucose-6-phosphate (G-6-P) [40]. HK has five isoforms—HK1-4 and a recently described hexokinase domain-containing protein 1 (HKDC1). HK2 is highly expressed in various cancers and is connected to poor pathological stage and prognosis [41, 42]. Since we found that the expression of HK2 decreased significantly after silencing or inhibiting the activity of NF-κBp65, the precise nature of the regulatory mechanism between NF-κBp65 and HK2 in HCC metabolism requires further investigation. We next performed luciferase and ChIP assays and found that NF-κBp65 can bind directly to the promoter of *HK2*. These results strongly suggest that NF-κBp65 dictates the metabolic phenotype in HBx-related HCC by regulating HK2.

The production of lactate from glucose metabolism represents one of the consequences of glycolysis. Cancer cells secrete lactate to provide an acidic pH in the tumour microenvironment (TME). Recently, an increasing number of studies have focused on the biological and metabolic molecular roles of lactate generated by cancer cells [43, 44]. It is widely acknowledged that this glycolytic metabolite is not a waste product but plays a pleiotropic role in tumour growth and metastasis. Numerous reports have revealed that lactate can function as a signalling molecule [28, 45]. Lactate activates the PI3K/Akt pathway to promote tumour angiogenesis in endothelial cells [28]. Lactate upregulates the expression of the antiapoptotic protein Bcl-2 via the PI3K/Akt/mTOR signalling pathway and promotes cell survival resistance to glucose starvation [45]. Consistent with previous studies, we found that lactate, but not pyruvate, promoted HCC cell proliferation via the MCT1/PI3K/Akt pathway. The pro-growth ability of lactate in HCC cells could be restrained by an MCT1 inhibitor or Akt inhibitor. These findings indicate potential therapeutic targets for HCC.

## Conclusions

In summary, this study demonstrates that HBx initiates the expression and activation of NF-κBp65 in hepatocytes and enhances aerobic glycolysis via the NF-κBp65/HK2 pathway to overproduce lactate, further increasing hepatocyte proliferation through PI3K/Akt signalling and resulting in hepatocellular carcinogenesis (Fig. 8i). Inhibition of NF-κBp65 in hepatocytes decreases the incidence of HBx-initiated HCC by downregulating aerobic glycolysis and pernicious proliferation. These findings suggest that phosphorylation of NF-κBp65 will be a potential therapeutic target for HBV-related HCC.

## Supplementary Information

**Additional file 1:** **Figure S1. **NF-κB family members in HCC. **a-e** The overall survival rates of NF-κBp65, RelB, cRel, NF-κB1 and NF-κB2 in HCC patients were analysed using the Gene Expression Profiling Interactive Analysis (GEPIA) online database. **f** The expression of *NF-κBp65 *mRNA in HBV-related HCC and normal liver tissues was analysed by real-time PCR (*n*=10 per group). Values are the mean ± SD. *P < 0.05 using Student’s t test. **g, h** The NF-κBp65 and p-p65 indices of 10 HBV-related HCC samples and 10 normal liver tissue samples were quantified by ImageJ software. Values are the mean ± SD. *P< 0.05, **P < 0.01 using Student’s t test. **Figure S2. **NF-κBp65 was associated with the poor prognosis in HCC patients. **a** Representative images of NF-κBp65 staining in HCC and paracancerous tissue microarrays. The expression level was evaluated according to the immunoreactivity score. **b, c** The maximal tumoursize (cm) in different NF-κBp65 expression level of HCC and paracanceroustissues in non-HBV-related HCC and HBV-related HCC. Values are the mean ± SD. *P*< 0.05 by Student’s t test. **d, e** Survival analysis in non-HBV-related HCC and HBV-related HCC with different NF-κBp65 expression. *P* < 0.05 by Kaplan-Meiersurvival analysis. **Figure S3.** HBx induced NF-κBp65 expression and phosphorylation in vitro and in vivo. **a** Quantification of HBx, NF-κBp65 and p-p65 IHC staining of liver tissues from WT and HBx-TG mice at 6 months and 18 months. Values are the mean ± SD (*n*=6 for each group). *P < 0.05 using Student’s t test. **b, c** Quantification of NF-κBp65 and p-p65 protein in LO2 and HepG2 cells transfected with vector, *pHBV 1.3* or *pHBV 1.3 x-null*. Values are the mean ± SD. *P < 0.05 compared with the vector group, ^#^*P*< 0.05 compared with the *HBV*-transfected group using one-way ANOVA. **d** Quantification of HBx and NF-κBp65 in LO2, HepG2, HepG2.2.15 and Hep3B cell lines. Values are the mean ± SD. *P < 0.05 compared with LO2 cells, ^#^*P *<0.05 compared with HepG2 cells using one-way ANOVA. **e** Quantification of HBx, NF-κBp65 and p-p65 protein in LO2 and HepG2 cells transfected with the *HA-HBx *or vector plasmid. **f** Quantification of NF-κBp65 and HBx protein in HepG2.2.15 and Hep3B cells transfected with the *flag-p65* or vector plasmid. **g** By incubation with HBV-infected patient serum, HepG2-NTCP cells were infected with HBV virions, and the level of HBV DNA in the cell supernatant was tested. The control group was incubated with healthy volunteers’ serum. **P < 0.01 using Student’s t test. **h, i** Western blotting analysis and quantification of NF-κBp65 and p-p65 protein in HepG2-NTCP cells infected with HBV virions. Values are the mean ± SD (*n*=3 for each group). *P < 0.05 using Student’s t test. **j** Quantification of the NF-κBp65 nuclear translocation index in LO2 and HepG2 cells stably transfected with the* HA-HBx* lentivirus and vector lentivirus. **k** Quantification of HBx, NF-κBp65 and p-p65 protein in the cytoplasm and nucleus in LO2 and HepG2 cells stably transfected with the *HA-HBx* lentivirus and vector lentivirus. Values are the mean ± SD. *P < 0.05 using Student’s t test. **l** *Flag-p65* and *HA-HBx *plasmids were transfected into HepG2 cells. Co-IP was used to detect the interaction between HBx and NF-p65 in HepG2 cells. **Figure S4.** HBx enhanced aerobic glycolysis in hepatocellular carcinogenesis. **a** The lactate content was measured in human normal liver tissues and HBV-related HCC tissues. *n*=6 per group. Values are the mean ± SD. *P < 0.05 using Student’s t test. **b, c** Quantification of HBx, GLUT1, HK2 and LDHA protein in LO2 and HepG2 cells transfected with *HA-HBx* or vector plasmid. Values are the mean ± SD. *P < 0.05 using Student’s t test. **Figure S5.** Inhibitionof glycolysis restrained HBx-induced proliferation. LO2 and HepG2 cells with or without stable expression of *HA-HBx* were treated with 2-DG (10 mM) for 24 h. **a, c** *PCNA* mRNA in different groups was detected by real-time PCR. **b, d** Western blot analysis of HBx and PCNA protein expression in cells and quantification of the relative PCNA protein expression. The experiment was repeated three times. All values are the mean ± SD. One-way ANOVA was used. *P < 0.05 compared with the vector group. ^#^*P *< 0.05 compared with the *HBx* group. **Figure S6.** HBx reprogramed glycolytic metabolism via NF-κBp65/HK2 signalling in hepatocellular carcinogenesis. **a, b** Quantification of NF-κBp65-Flag, GLUT1, HK2 and LDHA protein expression in HepG2.2.15 and Hep3B cells transiently transfected with *NF-κBp65* plasmids. **c, d** Quantification of NF-κBp65-Flag, GLUT1, HK2, LDHA and PCNA protein expression in HepG2.2.15 and Hep3B cellstransfected with *HK2* siRNA or combined with the *NF-κBp65 *plasmid. *P < 0.05 by one-way ANOVA. **e, f ** *HK2* mRNA levels in HepG2.2.15 and Hep3B cells transiently transfected with *NF-κBp65* plasmids or *NF-κBp65* siRNA. All values are the mean ± SD. *P < 0.05 by Student's t test. **Table S1.** Clinicopathological features in 10 HBV-related HCC cases and 10 liver haemangioma cases. **Table S2.** Clinicopathological features in 31 non-HBV-related HCC cases and 82 HBV-related HCC cases in tissue microarrays. **Table S3. **Primers of genes for quantitative PCR. **Table S4.** Primers of genes for ChIP‒qPCR.

## Data Availability

The data in the current study are available from the corresponding authors upon reasonable request.

## Abbreviations

HBV: Hepatitis B virus
HBx: Hepatitis B virus X protein
CHC: α-Cyano-4-hydroxycinnamate
ATP: Adenosine triphosphate
ChIP: Chromatin immunoprecipitation
2-DG: 2-Dexoxy-D-glucose
GLUT: Glucose transporter
HCC: Hepatocellular carcinoma
H&E: Haematoxylin and eosin
HK: Hexokinase
HKDC1: Hexokinase domain containing protein 1
IF: Immunofluorescence
IHC: Immunohistochemical
IκB: NF-κB inhibitor
IKK: IκB kinase
LDHA: Lactate dehydrogenase-A
MCT: Monocarboxylate transporter
mRNA: Messenger RNA
MEF: Mouse embryonic fibroblast
mTOR: Mechanistic target of rapamycin kinase
NF-κB: Nuclear factor kappa B
NEMO: NF-κB essential modulator
OXPHOS: Oxidative phosphorylation
PCNA: Proliferating cell nuclear antigen
PI3K: Phosphatidylinositol 3-kinase
PKB: Protein kinase B
TME: Tumour microenvironment
WT: Wild type
