# Supplementary material for: Aerobic glycolysis enhances HBx-initiated hepatocellular carcinogenesis via NF-κBp65/HK2 signalling
Source: J Exp Clin Cancer Res. 2022 Nov 21;41:329. doi: 10.1186/s13046-022-02531-x (PMC9677649; doi:10.1186/s13046-022-02531-x)
Supplement: Supplementary file 1 — Additional file 1: Figure S1. NF-κB family members in HCC. a-e The overall survival rates of NF-κBp65, RelB, cRel, NF-κB1 and NF-κB2 in HCC patients were analysed using the Gene Expression Profiling Interactive Analysis (GEPIA) online database. f The expression of NF-κBp65 mRNA in HBV-related HCC and normal liver tissues was analysed by real-time PCR (n=10 per group). Values are the mean ± SD. *P < 0.05 using Student’s t test. g, h The NF-κBp65 and p-p65 indices of 10 HBV-related HCC samples and 10 normal liver tissue samples were quantified by ImageJ software. Values are the mean ± SD. *P< 0.05, **P < 0.01 using Student’s t test. Figure S2. NF-κBp65 was associated with the poor prognosis in HCC patients. a Representative images of NF-κBp65 staining in HCC and paracancerous tissue microarrays. The expression level was evaluated according to the immunoreactivity score. b, c The maximal tumoursize (cm) in different NF-κBp65 expression level of HCC and paracanceroustissues in non-HBV-related HCC and HBV-related HCC. Values are the mean ± SD. P< 0.05 by Student’s t test. d, e Survival analysis in non-HBV-related HCC and HBV-related HCC with different NF-κBp65 expression. P < 0.05 by Kaplan-Meiersurvival analysis. Figure S3. HBx induced NF-κBp65 expression and phosphorylation in vitro and in vivo. a Quantification of HBx, NF-κBp65 and p-p65 IHC staining of liver tissues from WT and HBx-TG mice at 6 months and 18 months. Values are the mean ± SD (n=6 for each group). *P < 0.05 using Student’s t test. b, c Quantification of NF-κBp65 and p-p65 protein in LO2 and HepG2 cells transfected with vector, pHBV 1.3 or pHBV 1.3 x-null. Values are the mean ± SD. *P < 0.05 compared with the vector group, #P< 0.05 compared with the HBV-transfected group using one-way ANOVA. d Quantification of HBx and NF-κBp65 in LO2, HepG2, HepG2.2.15 and Hep3B cell lines. Values are the mean ± SD. *P < 0.05 compared with LO2 cells, #P <0.05 compared with HepG2 cells using one-way ANOVA. e Quantificat [file 13046_2022_2531_MOESM1_ESM.docx]

**SUPPLEMENTAL MATERIALS**

**Aerobic glycolysis enhances HBx-initiated hepatocellular carcinogenesis via NF-κBp65/HK2 signalling**

Lingjun Chen^1,2†^, Xianyi Lin^1,2†^, Yiming Lei^1,2^, Xuan Xu^1,2^, Qi Zhou^1,2^, Yan Chen^1,2^, Huiling Liu^1,2^, Jie Jiang^1,2^, Yidong Yang^1,2^, Fengping Zheng^1,2^ and Bin Wu^1,2, *^

^1^Department of Gastroenterology, The Third Affiliated Hospital of Sun Yat-Sen University, Guangzhou, Guangdong Province, 510630, China

^2^Guangdong Provincial Key Laboratory of Liver Disease Research, Guangzhou, Guangdong Province, 510630, China

Table of contents:

1. Supplemental Figure 1
2. Supplemental Figure 2
3. Supplemental Figure 3
4. Supplemental Figure 4
5. Supplemental Figure 5
6. Supplemental Figure 6
7. Supplemental Table 1
8. Supplemental Table 2
9. Supplemental Table 3
10. Supplemental Table 4
11. LO2 Cell STR identification report
12. HepG2 Cell STR identification report

**Figure S1. NF-κB family members in HCC.** (a-e) The overall survival rates of NF-κBp65, RelB, cRel, NF-κB1 and NF-κB2 in HCC patients were analysed using the Gene Expression Profiling Interactive Analysis (GEPIA) online database. (f) The expression of *NF-κBp65* mRNA in HBV-related HCC and normal liver tissues was analysed by real-time PCR (n=10 per group). Values are the mean ± SD. ******P* < 0.05 using Student’s t test. (g, h) The NF-κBp65 and p-p65 indices of 10 HBV-related HCC samples and 10 normal liver tissue samples were quantified by ImageJ software. Values are the mean ± SD. ******P* < 0.05, *******P* < 0.01 using Student’s t test.

**Figure S2. NF-κBp65 was associated with the poor prognosis in HCC patients.** (a) Representative images of NF-κBp65 staining in HCC and paracancerous tissue microarrays. The expression level was evaluated according to the immunoreactivity score. (b, c) The maximal tumour size (cm) in different NF-κBp65 expression level of HCC and paracancerous tissues in non-HBV-related HCC and HBV-related HCC. Values are the mean ± SD. *P* < 0.05 by Student’s t-test. (d, e) Survival analysis in non-HBV-related HCC and HBV-related HCC with different NF-κBp65 expression. *P* < 0.05 by Kaplan-Meier survival analysis.

**Figure S3.** **HBx induced NF-κBp65 expression and phosphorylation *in vitro* and *in vivo*.** (a) Quantification of HBx, NF-κBp65 and p-p65 IHC staining of liver tissues from WT and HBx-TG mice at 6 months and 18 months. Values are the mean ± SD (n=6 for each group). ******P* < 0.05 using Student’s t test. (b, c) Quantification of NF-κBp65 and p-p65 protein in LO2 and HepG2 cells transfected with vector, *pHBV 1.3* or *pHBV 1.3 x-null*. Values are the mean ± SD. ******P* < 0.05 compared with the vector group, **^#^***P* < 0.05 compared with the *HBV*-transfected group using one-way ANOVA. (d) Quantification of HBx and NF-κBp65 in LO2, HepG2, HepG2.2.15 and Hep3B cell lines. Values are the mean ± SD. ******P* < 0.05 compared with LO2 cells, **^#^***P* < 0.05 compared with HepG2 cells using one-way ANOVA. (e) Quantification of HBx, NF-κBp65 and p-p65 protein in LO2 and HepG2 cells transfected with the *HA-HBx* or vector plasmid. (f) Quantification of NF-κBp65 and HBx protein in HepG2.2.15 and Hep3B cells transfected with the *flag-p65* or vector plasmid. (g) By incubation with HBV-infected patient serum, HepG2-NTCP cells were infected with HBV virions, and the level of HBV DNA in the cell supernatant was tested. The control group was incubated with healthy volunteers’ serum. ***P* < 0.01 using Student’s t test. (h, i) Western blotting analysis and quantification of NF-κBp65 and p-p65 protein in HepG2-NTCP cells infected with HBV virions. Values are the mean ± SD (n=3 for each group). **P* < 0.05 using Student’s t test. (j) Quantification of the NF-κBp65 nuclear translocation index in LO2 and HepG2 cells stably transfected with the *HA-HBx* lentivirus and vector lentivirus. (k) Quantification of HBx, NF-κBp65 and p-p65 protein in the cytoplasm and nucleus in LO2 and HepG2 cells stably transfected with the *HA-HBx* lentivirus and vector lentivirus. Values are the mean ± SD. ******P* < 0.05 using Student’s t test. (l) *Flag-p65* and *HA-HBx* plasmids were transfected into HepG2 cells. Co-IP was used to detect the interaction between HBx and NF-p65 in HepG2 cells.

**Figure S4.** **HBx enhanced aerobic glycolysis in hepatocellular carcinogenesis.** (a) The lactate content was measured in human normal liver tissues and HBV-related HCC tissues. n=6 per group. Values are the mean ± SD. ******P* < 0.05 using Student’s t test. (b, c) Quantification of HBx, GLUT1, HK2 and LDHA protein in LO2 and HepG2 cells transfected with *HA-HBx* or vector plasmid. Values are the mean ± SD. ******P* < 0.05 using Student’s t test.

**Figure S5.** **Inhibition of glycolysis restrained HBx-induced proliferation.** LO2 and HepG2 cells with or without stable expression of *HA-HBx* were treated with 2-DG (10 mM) for 24 h. (a, c) *PCNA* mRNA in different groups was detected by real-time PCR. (b, d) Western blot analysis of HBx and PCNA protein expression in cells and quantification of the relative PCNA protein expression. The experiment was repeated three times. All values are the mean ± SD. One-way ANOVA was used. ******P* < 0.05 compared with the vector group. **^#^***P* < 0.05 compared with the *HBx* group.

**Figure S6.** **HBx reprogramed glycolytic metabolism via NF-κBp65/HK2 signalling in hepatocellular carcinogenesis.** (a, b) Quantification of NF-κBp65-Flag, GLUT1, HK2 and LDHA protein expression in HepG2.2.15 and Hep3B cells transiently transfected with *NF-κBp65* plasmids. (c, d) *HK2* mRNA levels in HepG2.2.15 and Hep3B cells transiently transfected with *NF-κBp65* plasmids or *NF-κBp65* siRNA. All values are the mean ± SD. ******P* < 0.05 by Student’s t test. (e, f) Quantification of NF-κBp65-Flag, GLUT1, HK2, LDHA and PCNA protein expression in HepG2.2.15 and Hep3B cells transfected with *HK2* siRNA or combined with the *NF-κBp65* plasmid. ******P* < 0.05 by one-way ANOVA.

**Table S1.** Clinicopathological features in 10 HBV-related HCC cases and 10 liver haemangioma cases

| Variable | HBV-related HCC  (n=10) | Liver haemangioma  (n=10) | *P* value |
| --- | --- | --- | --- |
| Age | 49.6 ± 9.7 | 51.3 ± 10.4 | 0.710 |
| Gender |  |  |  |
| Male | 8 (80.0%) | 7 (70.0%) | >0.9999 |
| Female | 2 (20.0%) | 3 (30.0%) |  |
| Tumour Size |  |  |  |
| ≤5 cm | 5 (50.0%) |  | - |
| >5 cm | 5 (50.0%) |  | - |
| Tumour grade |  |  |  |
| High | 4 (40.0%) |  | - |
| Moderate | 4 (40.0%) |  | - |
| Low | 2 (20.0%) |  | - |
| TNM stage |  |  |  |
| I | 1 (10.0%) |  | - |
| II | 5 (50.0%) |  | - |
| III & IV | 4 (40.0%) |  | - |

**Table S2.** Clinicopathological features in 31 non-HBV-related HCC cases and 82 HBV-related HCC cases in tissue microarrays

| Variable | Non-HBV-related HCC  (n=31) | HBV-related HCC  (n=82) | *P* value |
| --- | --- | --- | --- |
| Age | 57.9 ± 13.7 | 52.7 ± 10.0 | 0.029 |
| Gender |  |  |  |
| Male | 25 (80.6%) | 71 (86.6%) | 0.431 |
| Female | 6 (19.4%) | 11 (13.4%) |  |
| Tumour Size |  |  |  |
| ≤5 cm | 12 (38.7%) | 54 (65.9%) | 0.009 |
| >5 cm | 19 (61.3%) | 28 (34.1%) |  |
| Tumour grade |  |  | 0.37 |
| High | 6 (19.4%) | 26 (31.7%) |  |
| Moderate | 23 (74.2%) | 52 (63.4%) |  |
| Low | 2 (6.5%) | 3 (3.7%) |  |
| TNM stage |  |  | 0.098 |
| I | 16 (51.6%) | 59 (72.0%) |  |
| II | 10 (32.3%) | 13 (15.9%) |  |
| III & IV | 5 (16.1%) | 10 (12.2%) |  |

**Table S3. Primers of genes for quantitative PCR**

| **Gene** | **Orientation** | **Sequence (5’-3’)** |
| --- | --- | --- |
| *HBx* | F | TACCGTCCCTTGCTTTCTCT |
|  | R | CAGAGGTGAAGCGAAGTGC |
| *NF-κBp65* | F | ATGTGGAGATCATTGAGCAGC |
|  | R | CCTGGTCCTGTGTAGCCATT |
| *HK2* | F | TTC TTG GCC TTG GAC CTT G |
|  | R | CCA GAT GCC TTG AAG CCT TTT |
| *PCNA* | F | CAAGTAATGTCGATAAAGAGGAGG |
|  | R | GTGTCACCGTTGAAGAGAGTGG |
| *MCT1* | F | GGTGGAGGTCCTATCAGCAGT |
|  | R | CAGAAAGAAGCTGCAATCAAGC |
| *β-Actin* | F | GTCTTCCCCTCCATCGTG |
|  | R | AGGGTGAGGATGCCTCTCTT |

**Table S4. Primers of genes for ChIP‒qPCR**

| **Gene** | **Orientation** | **Sequence (5’-3’)** |
| --- | --- | --- |
| *NF-κBp65* | F | AGGGAAAACGGGGTAAGGAATC |
|  | R | AGGGGAGAACGCATCTGATTC |
| *HK2* | F | GCCTTGCCTCAATTTCCTCATC |
|  | R | AATACAAGAGTCGGCCCATCC |

**LO2 cell STR identification report**


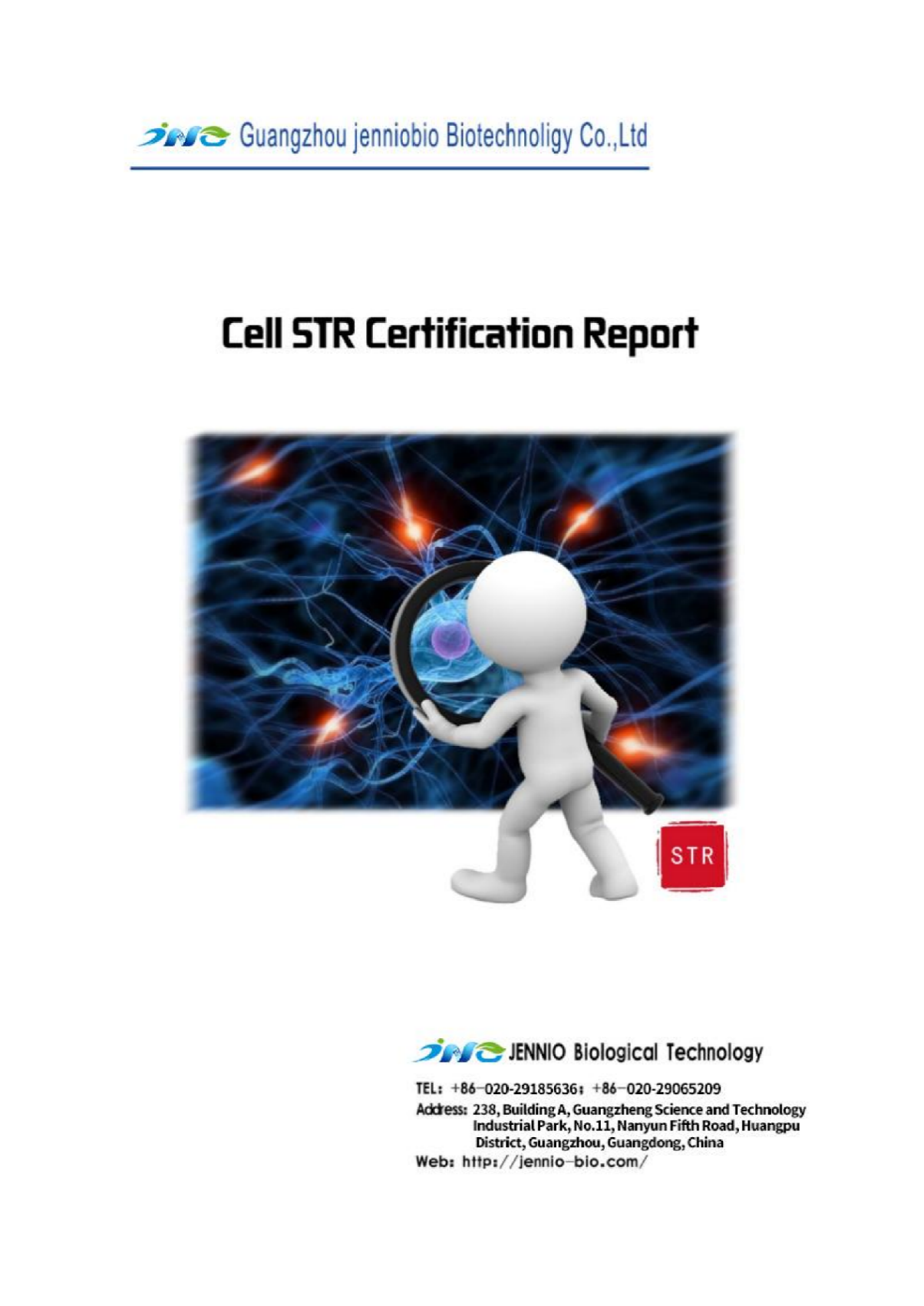

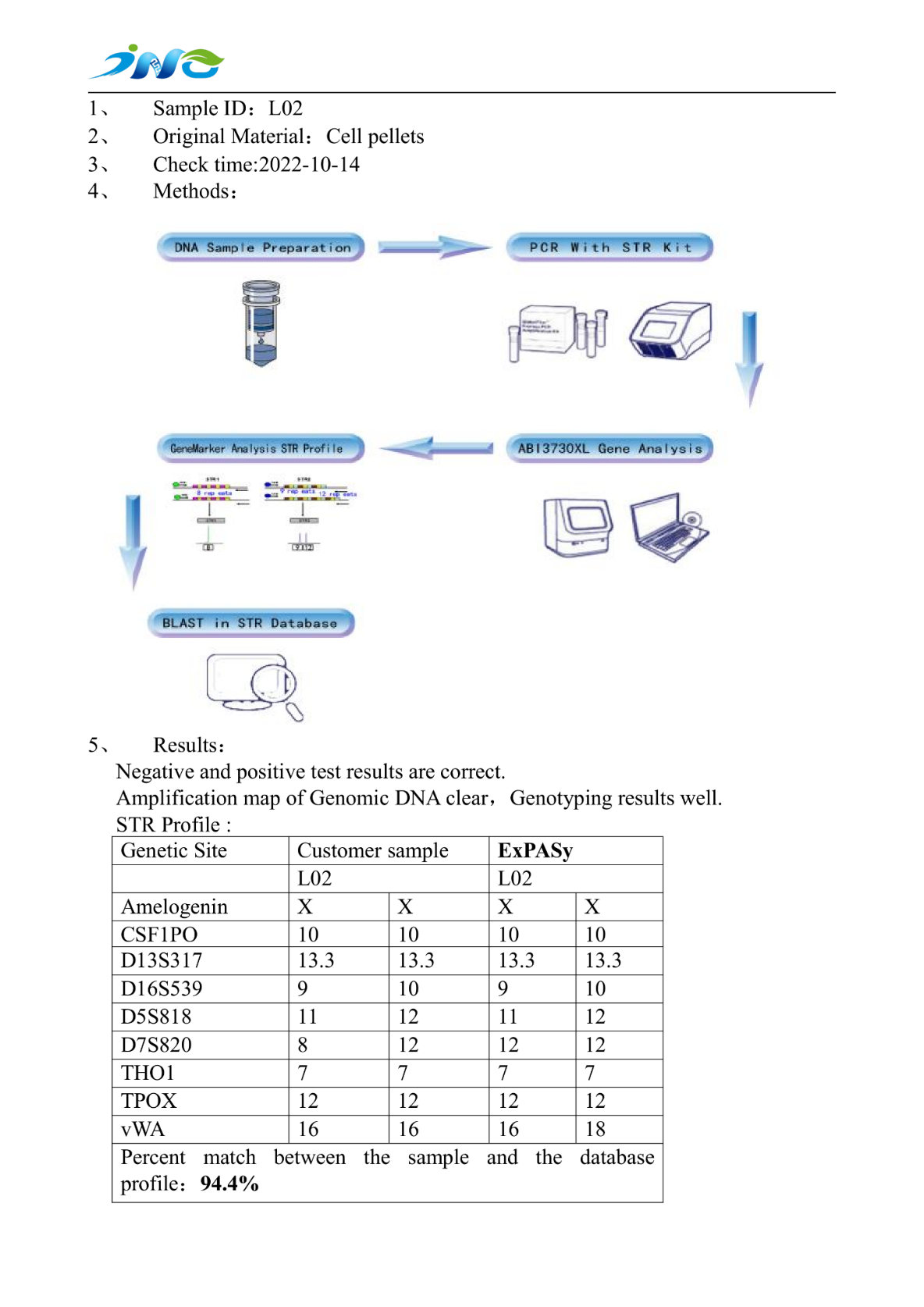

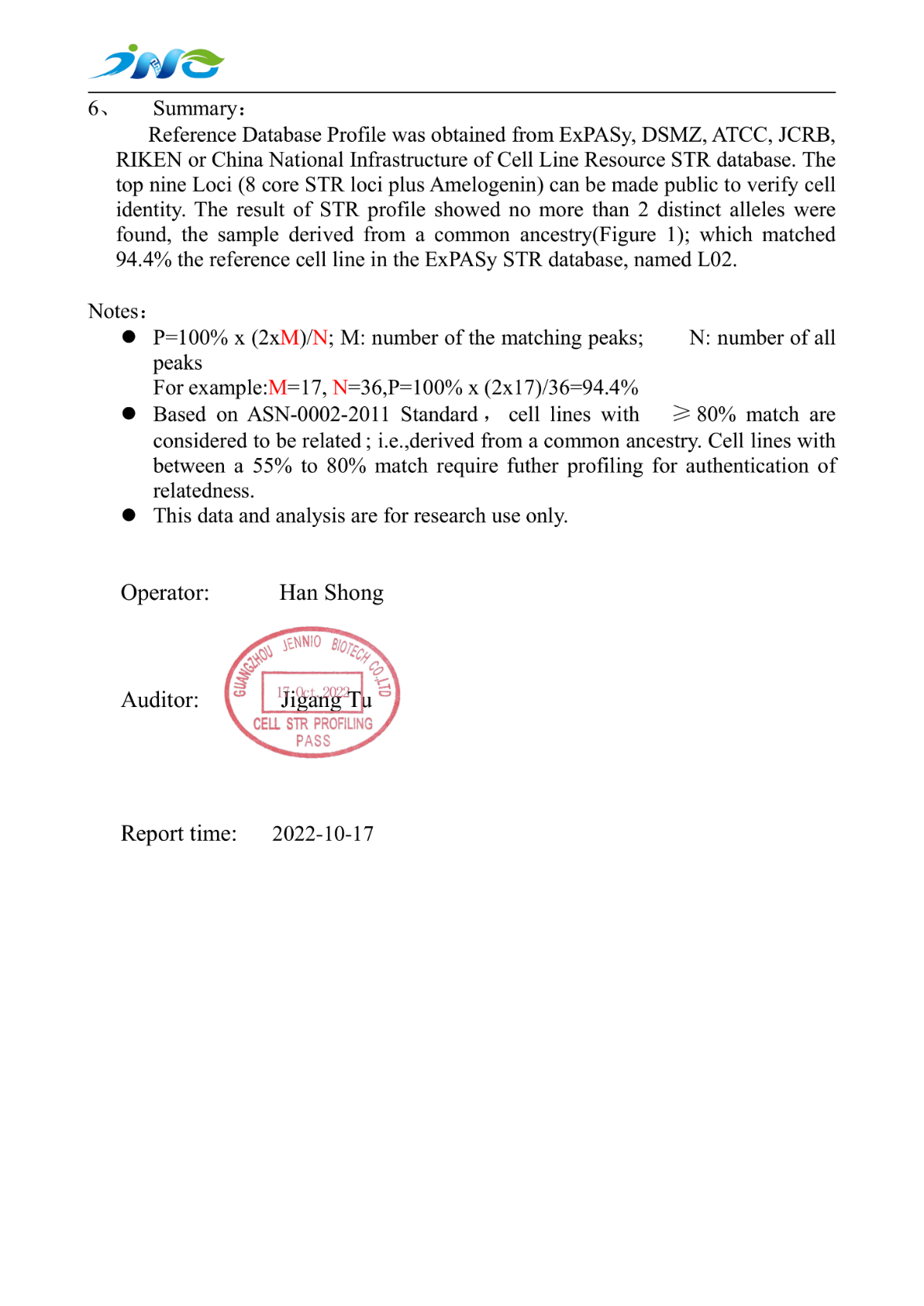

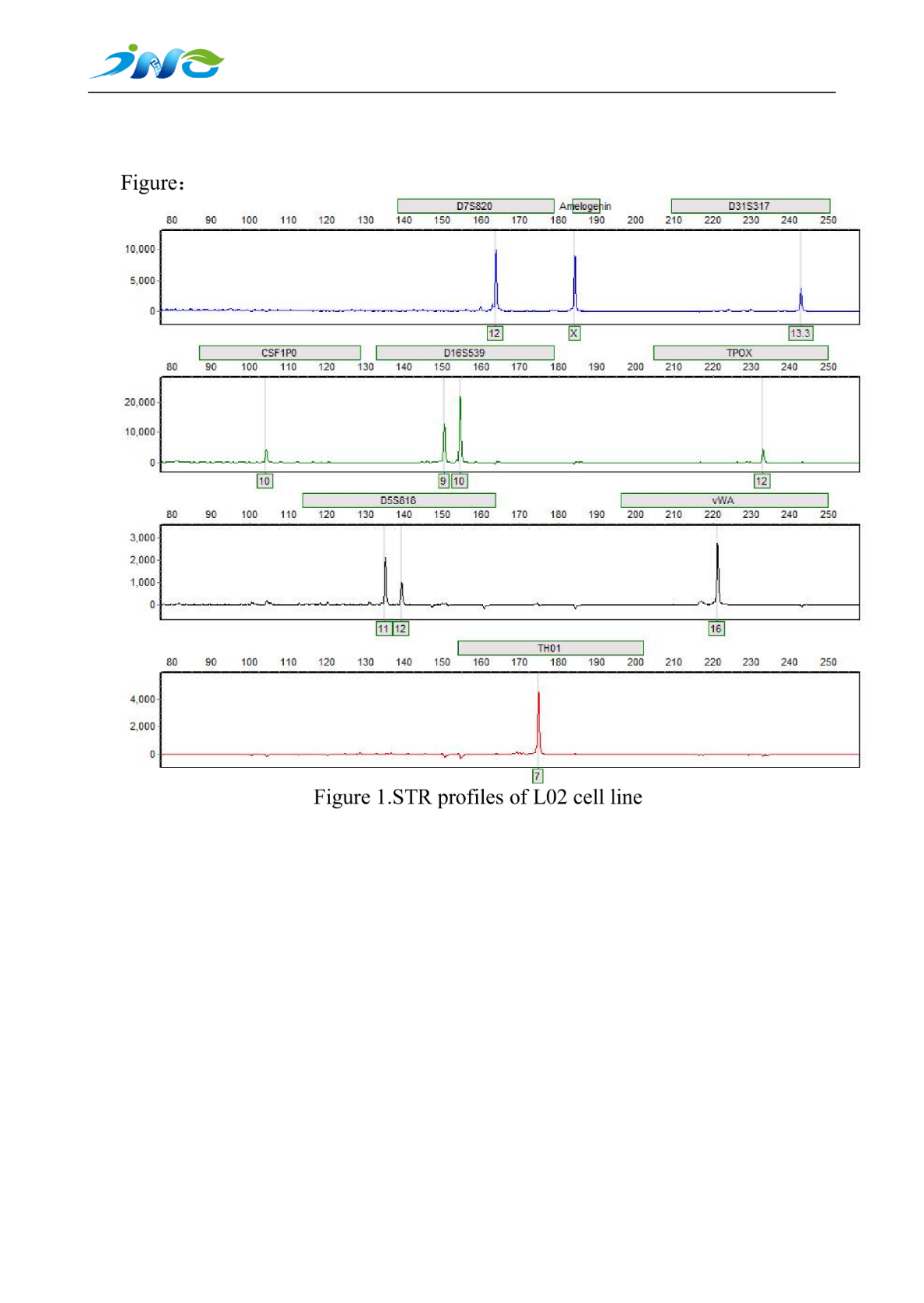


**HepG2 cell STR identification report**
